# Evolution of Maternal Provisioning and Development in the Ophiuroidea: Egg Size, Larval Form, and Parental Care

**DOI:** 10.1093/icb/icae048

**Published:** 2024-05-23

**Authors:** Maria Byrne, Paula Cisternas, Timothy D O'Hara, Mary A Sewell, Paulina Selvakumaraswamy

**Affiliations:** School of Life and Environmental Sciences and Marine Studies Institute, The University Sydney, Sydney, New South Wales 2006, Australia; School of Life and Environmental Sciences and Marine Studies Institute, The University Sydney, Sydney, New South Wales 2006, Australia; Museum Victoria, 11 Nicholson St, Melbourne, Victoria 3001, Australia; Museum Victoria, 11 Nicholson St, Melbourne, Victoria 3001, Australia; School of Biological Sciences, University of Auckland, Auckland, New Zealand; School of Life and Environmental Sciences and Marine Studies Institute, The University Sydney, Sydney, New South Wales 2006, Australia

## Abstract

The Ophiuroidea is the most speciose class of echinoderms and has the greatest diversity of larval forms, but we know less about the evolution of development (evo-devo) in this group than for the other echinoderm classes. As is typical of echinoderms, evo-devo in the Ophiuroidea resulted in the switch from production of small eggs and feeding (planktotrophic) larvae to large eggs and non-feeding (lecithotrophic) larvae. Parental care (ovoviviparity or viviparity/matrotrophy) is the most derived life history. Analysis of egg data for 140 species (excluding viviparity and facultative planktotrophy) indicated a bimodal distribution in egg volume corresponding to planktotrophy and lecithotrophy + ovoviviparity, with three significant egg size groups due to the very large eggs of the ovoviviparous species. The marked reduction in fecundity in species with extremely large eggs is exemplified by the ovoviviparous species. Egg size in the two species with facultative planktotrophy was intermediate with respect to the two modes. Identifying the ancestral larval life history pattern and the pathways in the switch from feeding to non-feeding larvae is complicated by the two patterns of metamorphosis seen in species with planktotrophic development: Type I (ophiopluteus only) and Type II (ophiopluteus + vitellaria larva). The variability in arm resorption at metamorphosis across ophiuroid families indicates that the Type I and II patterns may be two ends of a morphological continuum. This variability indicates ancestral morphological plasticity at metamorphosis, followed by canalization in some taxa to the vitellaria as the metamorphic larva. Vestigial ophiopluteal traits in lecithotrophic ophioplutei and vitellaria indicate evolution from the ancestral (feeding larva) state. Parental care has evolved many times from an ancestor that had a planktonic ophiopluteus or vitellaria and is often associated with hermaphroditism and paedomorphosis. A secondary reduction in egg size occurred in the viviparous species.

## Introduction

Life history traits in marine invertebrates, such as the contrast between broadcast spawning and parental care or the possession of feeding or non-feeding larvae, have a profound influence on species’ population biology, ecology, population genetics, and biogeography (Thorson 1950; Pearse 1994; Havenhand 1995; Podolsky and Strathmann 1996; Puritz et al. 2017; Klanten et al. 2023). Life history traits across development, from eggs to larvae and the benthic juvenile, typically reflect the extent of maternal provisioning. Species with small eggs tend to have feeding larvae (= planktotrophy) and a long pelagic period with the potential for long dispersal (Shanks 2009). In contrast, species with larger eggs generally have non-feeding larvae (= lecithotrophy) and a short pelagic period, or, in species that care for their young to the juvenile stage, no or very limited dispersal (Strathmann 1985, 1993; Byrne 1991; Jaeckle 1995; Raff and Byrne 2006; Byrne et al. 2008; Khan et al. 2020). The contrast between the high fecundity-small egg-planktotrophic larvae and the low fecundity-large egg-lecithotrophic larval life history modes has received extensive ecological and theoretical consideration (Vance 1973; Strathmann 1985, 1993; Havenhand 1995; Jaeckle 1995; Hart 1995; Podolsky and Strathmann 1996; McEdward 1997, 2000; McEdward and Miner 2001; Moran and McAlister 2009; Prowse et al. 2008, 2009; Allen 2012; Marshall et al. 2012; Moran et al. 2013).

The Echinodermata has a high diversity of larval forms across the spectrum of planktotrophy and lecithotrophy including facultative feeding larvae, as well as vestigial larvae provisioned by matrotrophy (Strathmann 1985; McEdward and Miner 2001; Byrne 2006; Raff and Byrne 2006; Allen and Podolsky 2007; Mercier et al. 2013; Hodin et al. 2019; Khan et al. 2020). This diversity has been used as a model system to test hypotheses on life history evolution (Wray 1996, 2022; Hart et al. 1997; Raff and Byrne 2006). For echinoderms, the production of a small egg and development through a planktotrophic larva is considered the plesiomorphic condition, with large eggs and non-feeding development arising independently several times (Wray 1996; McEdward and Miner 2001; Raff and Byrne 2006; Keever and Hart 2008; Arnone et al. 2015; Hodin et al. 2019). Although evidence for the evolutionary transition (e.g., smaller eggs to bigger eggs) is suggested to be equivocal based on parsimony, an assessment of parsimony assumptions countering this suggestion is critiqued in Keever and Hart (2008).

Based on egg diameter, a bimodal egg size distribution along the planktotrophy-lecithotrophy dichotomy is seen in the Asteroidea and Echinoidea and, to a lesser extent, in the Holothuroidea (Emlet et al. 1987; Jaeckle 1995; Sewell and Young 1997; McEdward and Miner 2001). For the Ophiuroidea, a unimodal egg size distribution is reported (Sewell and Young 1997). Modification of life history traits (egg size, larval nutrition), often within a relatively short evolutionary time, has been important in generating biological novelty in echinoderms (Wray 1996, 2022; Raff and Byrne 2006; Davidson et al. 2022).

Evo-devo research on echinoderms has primarily focused on asteroids and echinoids (Wray 1996, 2022; Hart et al. 1997; Byrne 2006; Raff and Byrne 2006; Arnone et al. 2015). Despite the Ophiuroidea having the highest species diversity of the echinoderm classes with ∼ 2100 described species (O'Hara et al. 2017; Stöhr et al. 2021) and the most diverse range of larval forms (McEdward and Miner 2001; Byrne and Selvakumaraswamuy 2002), there are fewer evo-devo studies of ophiuroids (Selvakumaraswamy and Byrne 2004, 2006, 2023; Allen and Podolsky 2007; Byrne et al. 2024). As is typical of echinoderms, loss of the ophiopluteus resulted in loss of feeding structures (e.g., larval arms, digestive tract) (Selvakumaraswamy and Byrne 2000a; Fourgon et al. 2005; Selvakumaraswamy and Byrne 2006, 2023). Several recent studies provide new information on ophiuroid egg size and larval development (see Supplementary Table S1), providing the opportunity to revisit egg and larval evolution in these echinoderms.

While the basic dichotomy of feeding vs non-feeding larvae holds for ophiuroids, development can also be characterized according to the phenotype of the larva at metamorphosis (Mortensen 1921, 1931; Mladenov 1985; Byrne and Selvakumaraswamy 2002; Selvakumaraswamy and Byrne 2006, 2023; Byrne et al. 2024). For species with feeding ophioplutei, Mortensen (1921) first recognized differences in the patterns of ophiopluteal arm resorption and rearrangements of ciliated bands at metamorphosis. Mladenov (1985) later defined these as two larval patterns. Some planktotrophic species have just the ophiopluteus (Type I development = one larval stage), while in others, the ophiopluteus transforms into a second armless larva, the vitellaria at metamorphosis (Type II development = two larval stages) (Mladenov 1985). This was based on the ophiopluteus-to-vitellaria transformation in an ophiocomid species (Mladenov 1985).

Species with feeding ophioplutei (planktotrophy) that have Type I development metamorphose in the plankton with the juvenile supported by the two remaining arms (posterolaterals). In some species, it appears that the posterolaterals are discarded while in the plankton or post-settlement (e.g., *Ophiopholis aculeata; Ophiothrix spongicola*), while, in others one or both of these arms are resorbed at metamorphosis (e.g., *Ophiodaphne formata, Ophiothrix ciliaris*) (Tominaga et al. 2004; Selvakumaraswamy and Byrne 2006; Kitazawa et al. 2014). The retention of the posterolaterals and their ciliary tract is suggested to provide the juvenile brittle star with mobility control while still in the plankton and during the presettlement exploration phase (Tominga et al. 2004). Species with non-feeding ophioplutei (lecithotrophy, e.g., *Macrophiothrix*) also have Type I development, and, in this case, development is through a yolky larva with a reduced number of arms (Fenaux 1963; Allen and Podolsky 2007). Regardless of when and where the larva discards or resorbs the last pair of larval arms, in ophiuroids with Type I development, the juvenile is fully metamorphosed at settlement and attaches with their tube feet (TF), and the ophiopluteus does not transform into a vitellaria (Selvakumaraswamy and Byrne 2006).

In species with feeding ophioplutei that have Type II development, all the larval arms are resorbed, and the ciliated band transforms into ciliary tracts in development of a second (armless) larva, the vitellaria. Thus far, this has only been seen for the Ophiocomidae (e.g., *Breviturma dentata, Ophiocomella pumila*). Metamorphosis is completed in the benthos in larvae attached with the juvenile podia (Brooks and Grave 1899; Cisternas and Byrne 2005; Byrne et al. 2024). Some ophiuroid families with lecithotrophic development (e.g., Ophiodermatidae) have only one larval form, the vitellaria (Grave 1900; Mortensen 1921, 1938; Fenaux 1963; Stancyk 1973; Hendler 1982; Komatsu and Shosaku 1993; Cisternas and Byrne 2005; Fourgon et al. 2005; Byrne et al. 2008; Sweet et al. 2019). The differences in the larval form at metamorphosis (Type I vs Type II) have prompted discussion about the ancestral pattern of larval development in the Ophiuroidea (Mladenov 1985; McEdward and Miner 2001; Selvakumaraswamy and Byrne 2006, 2023; Byrne et al. 2024).

Most ophiuroids that do not develop through a dispersive larva care for their young in the respiratory bursae, where they develop to the advanced juvenile, eventually leaving the parent through the bursal slits (Hendler 1975, 1991; Byrne 1991). Some species have benthic development, hatching as juveniles (Fell 1941; Hendler 1977; Schoppe and Holl 1994; Emlet 2006), and others incubate their young in the gonads (Turner and Dearborn 1979). Parental care is a derived life history mode in ophiuroids and is generally associated with small adult body size, hermaphroditism, and very large eggs (ovoviviparity) or small eggs and extraembryonic (viviparity-matrotrophy) nutrition (Mortensen 1920; Byrne 1991; Hendler 1991). In matrotrophic ophiuroids, the eggs are secondarily reduced in size, and the parent provides extra nutrients through the hemal system, other body fluids, nurse eggs, or potential cannibalism of clutch mates (Fell 1946; Hendler 1975; Turner and Dearborn 1979; Landschoff and Griffiths 2015).

In the decades following Hendler's seminal reviews (Hendler 1975, 1991), we have a greater understanding of development across ophiuroid families in studies that have linked egg size and biochemical composition to larval type (Selvakumaraswamy and Byrne 2000a,b, 2004, 2006; Byrne and Selvakumaraswamy 2002; Cisternas et al. 2004; Cisternas and Byrne 2005; Fourgon et al. 2005; Allen and Podolsky 2007; Whitehill and Moran 2012; Kitazawa et al. 2014; Falkner et al. 2013, 2015; O'Hara et al. 2019). With these data and the recent phylogenetic revision of the Class Ophiuroidea (O'Hara et al. 2017), we provide an evo-devo perspective of egg and larval evolution in these ecologically important echinoderms. For species with planktonic larvae, we generated data on eggs and/or larvae for nine species. For five ovoviviparous species that have very large eggs, we determined reproductive output (fecundity) and the relationship between fecundity and adult size. We expected that the maximum egg size trait seen in these species would strongly influence the egg size mode distribution. As the pattern of larval arm loss (discarded or resorbed) is variable, yet key to understanding ophiuroid larval evolution, we reared several species to the juvenile stage and considered the ancestral pattern of larval development.

## Materials and methods

### Species with planktonic larvae

Six species were collected by snorkel or SCUBA (1–20 m depth) to obtain new data on egg size and for spawning trials. Three species (*Ophioderma appressum, O. brevicaudum*, and *O. cinereum*) were collected from Galeta, Panama (9°N, 79°W), two (*Ophiopteris papillosa, Ophiothrix spiculata*) were collected from Monterey, California (36°N, 122°W), and *Ophiopsila californica* was collected from Santa Barbara (36°N, 122°W) California. Two species from the Museum Victoria (MV) collections were examined in gravid-preserved material: *Clarkcoma australis* (MV_F101798) and *Clarkcoma bollonsi* (MV_595683). *Ophiopsila pantherina* was collected from the continental shelf (70 m depth) at Hydrographers Passage (20°S, 150°E), Great Barrier Reef (Woolsey et al. 2013), and the gonads of this species were examined in preserved material. Spawning could not be induced in *Ophiopsila californica*, so we dissected the ovaries of freshly collected specimens to measure the eggs.

Eight species were collected for anatomy, histology, spawning, and/or larval rearing. These included: *Ophiocoma scolopendrina, Ophiomastix elegans*, and *Breviturma dentata* collected in shallow water (1–2 m depth) at One Tree Island (23°S, 152°E) and Lizard Island (14°S, 145°E) on the Great Barrier Reef; *Amphipholis pugetana* collected (20 m depth) in Barkley Sound, British Columbia (49°N, 125°W); *Ophiomastix endeani* collected intertidally near Coffs Harbour, New South Wales (30°S, 153°E); *Ophiactis resiliens, Clarkcoma pulchra*, and *Ophiarachnella ramsayi* collected from shallow water (1–2 m depth) at Clovelly Bay (34°S, 151°E) and Little Bay (33°S, 151°E), Sydney.

The ovaries of *Breviturma dentata* and *Ophiomastix endeani* were fixed in Bouin’s fluid and the tissue was processed using routine methods for wax histology. The sections (6 µm thick) were stained using the alcian blue—periodic acid Schiff's reagent method, which stains echinoderm yolk magenta (Byrne 1988; Byrne et al. 2003). The ovaries of these species were also fixed in cold 2.5% glutaraldehyde in 0.1 M cacodylate containing 0.01 g/mL NaCl, rinsed in cacodylate buffer, and post-fixed in 1% osmium tetroxide, followed by routine embedding for 1.0 µm plastic sections that were stained with toluidine blue.

### Spawning and rearing of larvae

Spawning was induced using the heat shock method (Selvakumaraswamy and Byrne 2000b). Most female ophiuroids cannot be induced to spawn in isolation, so spawning was induced in groups of two or more females in the presence of a male. The diameter of recently fertilized spawned eggs (*n* = 20–60 eggs per spawn) was measured using an ocular micrometer, and the mean egg diameter was determined. In each case, examination of unfertilized eggs indicated that fertilization had minimal/no effect on egg size. Egg buoyancy was noted. Fertilized eggs were washed free of sperm in filtered seawater (FSW, 1.0 µm Millipore) and placed in beakers (200–1000 mL, depending on the number of eggs). The FSW was renewed by reverse filtration daily. To connect egg size and developmental mode, we reared eight species. The three *Ophioderma* species were reared at 27°C; *Ophiothrix spiculata* and *Ophiopteris papillosa* were reared at 12°C; and the larvae of *C. pulchra, Ophiarachnella ramsayii*, and *O. resiliens* were reared at 18–20°C. To illustrate larval types, *O. resiliens* (Type I planktotroph), *C. pulchra*, and *Ophioderma cinereum* (latter two species Type II lecithotrophs), were reared to metamorphosis. Larvae were photographed with a dissecting microscope with a digital camera. Some larvae were processed for scanning electron microscopy following routine methods (see Selvakumaraswamy and Byrne 2004, 2023).

### Ovoviviparous and viviparous species

For the ophiuroids that care for their young, only the egg sizes of the ovoviviparous species were used in the egg-size analysis. Five ovoviviparous species that care for their young in the respiratory bursae were collected from Conch Key, Florida (25°N, 81oW) and Carrie Bow Cay on the Mesoamerican Barrier Reef, Belize (17°N, 88°W). *Ophionereis olivacea* (*n* = 137, 2.8–5.0 mm disc diameter- DD) and *Ophiolepis paucispina* (*n* = 188, 3.6–6.2 mm DD) were collected from clumps of *Halimeda opuntia* (0.5–1.1 m depth) from Conch Key monthly from January to December 1985 and from Twin Cays, Carrie Bow Cay, and Blue Ground Range, Belize, in November 1983, June 1985, and April 1986. *Amphiura stimpsonii* ( *n* = 33, 1.8–4.0 mm DD) and *Ophiurochaeta littoralis* (*n* = 105, 2.0–4.8 mm DD) were also collected through the use of snorkel or SCUBA from *H. opuntia* (2–5 m depth) on the same occasions (1983–1986), and *Sigsbea conifera* (*n* = 48, 2.8–6.4 mm DD) were collected using SCUBA from its host hydrocoral, *Stylaster roseus*, in the Carrie Bow Cay spur and groove fore-reef zone (5–10 m depth). *Amphipholis squamata*, was collected from shallow water (1–2 m depth) at Little Bay (33°S, 151°E), Sydney.

Disc diameters were measured with a ruler, and the bursae examined for the presence of juveniles and mature ovaries. The aboral side of the parent’s disc was carefully removed by cutting along its edge with scissors and severing the connective tissue that connects the disc to the skeletal elements above each arm to access the gonads and offspring. The bursae positioned at the base of each arm were gently teased open with fine forceps to remove the embryos and juveniles, which were counted. The eggs in fully mature ovaries were counted. For some species, this involved gently removing the eggs from the ovaries with fine forceps. Egg buoyancy was noted. The size of the largest eggs in the ovaries was measured with an ocular micrometer to indicate terminal egg size. The number of eggs in the gonads and young in the bursae were totaled to estimate fecundity, which was plotted with respect to the parent's disc diameter. For *O. olivacea*, which has a synchronous/annual gametogenic cycle followed by synchronous development of progeny (Byrne 1991), the number of eggs in the ovary and the number of young in the bursa were plotted separately. *Amphiura stimpsoni, Ophiurochaeta littoralis, Ophiolepis paucispina*, and *Sigsbea conifera* have asynchronous reproduction with advanced eggs in the gonads and offspring in the bursae simultaneously present for much of the year. In these species, the number of advanced eggs and embryos was combined to estimate fecundity.

The ovaries of *Ophiolepis paucispina* and *Sigsbea conifera* and the entire disc of *Amphipholis squamata* were fixed and decalcified in Bouin’s fluid, and the tissues were processed for wax histology. Sections (6 µm thick) of *Ophiolepis paucispina* and *Sigsbea conifera* were stained using the alcian blue—periodic acid Schiff’s reagent method, while the sections of *Amphipholis squamata* were stained using hematoxylin and eosin.

### Egg size data

The new egg size data from this study were combined with data from the literature (Supplementary Table S1) to analyze size trends (see below) in species with planktotrophic or lecithotrophic development. For the egg-size analysis, we focused on two categories where: (1) egg nutrients are fully utilized in formation of the larva, which then relies on exogenous food to develop to the juvenile (planktotrophy); and (2) egg nutrients fully support development to the juvenile (lecithotrophy) (see Supplementary Table S1 for species and data). We thus avoided the confounding influence of extra-embryonic nutrition. The analysis did not include egg size data for viviparous-matrotrophic species (listed in Supplementary Table S2). Data for the two species known to have facultative planktotrophy, *Macrophiothrix rabdota* and *Amphiodia* sp. (Allen and Podolsky 2007; Nakata and Emlet 2023) were also not included in the egg-size analysis as their development does not fall into the separate categories used (Supplementary Table S2). However, the egg sizes for these two species are illustrated in the histogram in the supplementary material to show where they are positioned with respect to the overall egg size distribution (Supplementary Fig. S1).

For species that there are no data on the larva, the presence of gravid ovaries with 1000’s of small eggs (≤ 150 µm diameter) was used to designate planktotrophy, while those with 100’s of large eggs (≥200 µm diameter) and with no evidence of young in the bursae were used to designate lecithotrophy (see Hendler 1991). These egg size categories reflect the egg sizes of species with known development (Supplementary Table S1). Overall, species with eggs between 150 and 200 µm were not present. Species that care for their young were identified by the presence of offspring in the bursa. In some cases where larvae were in the bursae, the type of larvae was identified (e.g., Byrne 1991; Byrne et al. 2008).

**Fig. 1:**
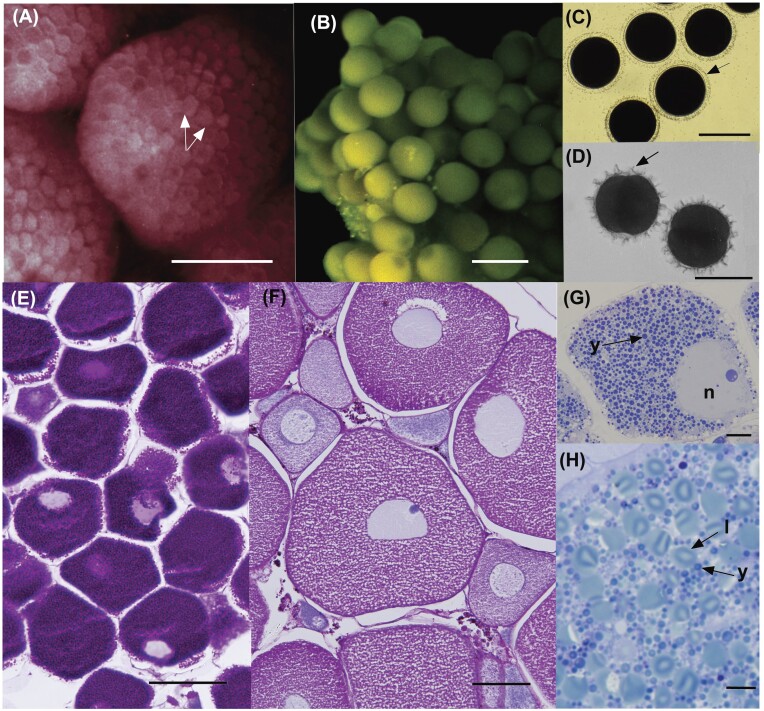
Mature ovaries and spawned eggs. (A) Ovaries in the planktotrophic developer, *Ophiocoma scolopendrina*, packed with small (100 µm diam.) red eggs (arrows). (B) Dissected gonads from *Ophiarthrum elegans* showing large (ca. 380 µm diam) green eggs of this lecithotrophic developer. (C, D) Fertilized eggs of *Breviturma dentata* (C) and *Ophiocoma scolopendrina* (D) with smooth and ornate fertilization envelopes (arrows), respectively. (E, F) Histology sections of the ovaries of *Breviturma dentata* (E) and *Ophiomastix endeani* (F). The magenta stain indicates presence of yolk (see methods), which dominates the eggs of *B. dentata*. (G, H) Plastic sections of the eggs of these species showing the dominance of yolk granules (Y) in the eggs of *B. dentata* (G) and lipid droplets (L) in the eggs of *O. endeani* (H). N, nucleus. Scale bars: A, B = 500 µm, C, D, F = 100 µm, E = 50 µm, G, H = 10 µm.

Egg size data (volume) from 140 species were used for analysis, not using data for the three of the species where the ovaries were dissected, but for which we could not discern terminal egg size (see Supplementary Table S1). Egg volume is a convenient measure of egg size as a proxy to assess the level of maternal provisioning, although egg biochemistry is better, albeit data are limited (Turner and Lawrence 1979; Moran and McAlister 2009; Falkner et al. 2015). As spawned eggs of ophiuroids are round (Fig. 1C, D), volume was calculated based on the formula for the volume of a sphere (4π r^3^/3) and is expressed in nanoliter (nL) (Supplementary Tables S1, S2).

### Statistical analyses

The egg volume data (ln-transformed) were analyzed to identify the number of modes (mixture components) in a Gaussian finite mixture model in the R package “mclust” (Scrucca et al. 2016). This approach assumes that modes are from a Gaussian distribution, and an inspection of the ln-transformed egg volume data for each development mode revealed approximate normality. The mixture model was fitted with the expectation-maximization algorithm and the optimum model selected using Bayesian Information Criteria.

As the Gaussian distribution indicated an overall bimodal distribution (planktotrophy-lecithotrophy), with egg size of the ovoviviparous species on the right side of the distribution, we analyzed the egg volume data with a one-way analysis of variance (ANOVA) with mode of development (planktotrophic planktonic larva, lecithotrophic planktonic larva, ovoviviparous) as a fixed factor. The raw egg volume data (nL) were ln transformed prior to analysis to achieve normality as indicated by Shapiro–Wilks test, but homogeneity of variance was not met as indicated by Levene’s test, so we used a Welch-corrected ANOVA to analyze the data. A Games–Howell post hoc test was used to assess statistical differences. These analyses were done with “rstatix” (R Core Team 2023). The egg and embryo count data for the ovoviviparous species across the size range of adults dissected were analyzed by linear regression with the R package “stats” (R Core Team 2023).

## Results

### Spawning, egg size, anatomy, and histology

Spawned eggs of the three *Ophioderma* species had mean diameters of 286 µm (SE = 2.3, *n* = 45), 273 µm (SE = 2.0, *n* = 40), and 348 µm (SE = 5.5, *n* = 52) for *Ophioderma appressum, O. brevicaudum*, and *O. cinereum*, respectively. These eggs were positively buoyant and green in color. Mean diameters of the eggs of *Ophiopteris papillosa* and *Ophiothrix spiculata* were 99 µm (SE = 0.9, *n* = 42), 110 µm (SE = 0, *n* = 20), respectively. These eggs were negatively buoyant. Eggs dissected from the gonads of *Clarkcoma australis, C. bollonsi, Ophiopsila californica*, and *O. pantherina* were 96 (SE = 0.1, *n* = 20), 100 µm (SE = 0, *n* = 20), 220 µm (SE = 1.8, *n* = 20), and 382 µm diameter (SE = 1.5, *n* = 24), respectively.

Examination of the ovaries and spawned eggs shows the contrast between the small eggs of the species with planktotrophic development and large eggs of the species with lecithotrophic development (Fig. 1A–H). Species with planktotrophic larvae spawn 1000’s of negatively buoyant eggs, while the species with lecithotrophic larvae spawn fewer (< 1000) eggs that are neutral or positively buoyant (see Falkner et al. 2015; O’Hara et al. 2019). This dichotomy is also reflected in egg histology. The small negatively buoyant eggs of *Breviturma dentata* had a homogeneous and intense PAS+ staining reaction, indicating the dominance of yolk protein (Fig. 1E, G). In contrast, the PAS+ response of the large eggs of *Ophiomastix endeani* was less intense due to the abundance of lipid deposits in the cytoplasm interspersed with yolk granules (Fig. 1F, H). This lipid contributes to their positive buoyancy. As is typical of echinoderms, the fertilized eggs of most ophiuroids have a smooth fertilization envelope (Fig. 1C). *Ophiocoma scolopendrin*a, by contrast, has an ornate fertilization envelope that has spikey projections (Fig. 1D) (see Mortensen 1937).

### Parental care

**Fig. 2:**
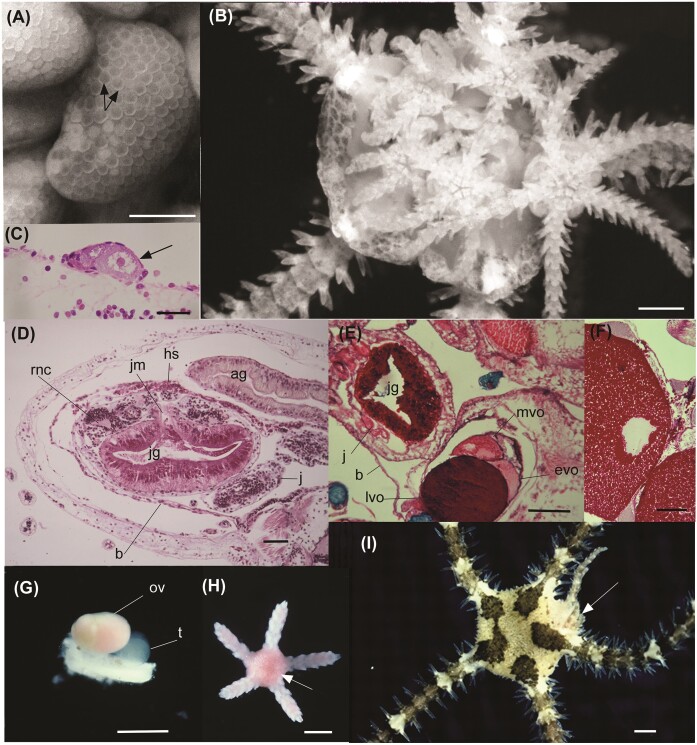
(A) Mature ovary of *Amphipholis pugetana* filled with eggs (arrows). (B) Dissected *Amphipholis squamata* showing large juveniles that were removed from the bursa. (C) Ovary of *A. squamata* with a single egg (arrow) developing. (D) Section of an adult *A. squamata*, aboral side up showing a large juvenile in the bursa (b) with PAS+ material (arrows) in the juvenile gut (JG) in the stomach wall and lumen, likely sourced from nutritive material in the hemal sinus (hs) taken up through the juvenile mouth (JM). The arms are well developed, as indicated by the radial nerve cord (RNC). The adult gut (AG) and a second juvenile (J) are also evident. (E) Section of an adult *Ophiolepis paucispina* showing a large juvenile (J) in the bursa (B) with PAS+ egg material in the JG and an ovary with late (LVO), mid (MVO), and early (EVO) vitellogenic oocytes as indicated by the different levels of PAS+ response. (F) Ovary of *Sigsbeia conifera* with large PAS+ oocytes. (G) Dissected genital plate of *O. paucispina* showing adjacent ovary (OV) and testis (T). (H) Juvenile *S. conifera* removed from the bursa with pink egg nutrients in the gut (arrow). (I) *Ophiurochaeta littoralis* with a large juvenile (arrow) leaving the bursa with the arm emerging first. Scale bars: A, B, G, H = 500 µm; C = 50 µm; D-F = 100 µm; I = 1 mm.

**Fig. 3:**
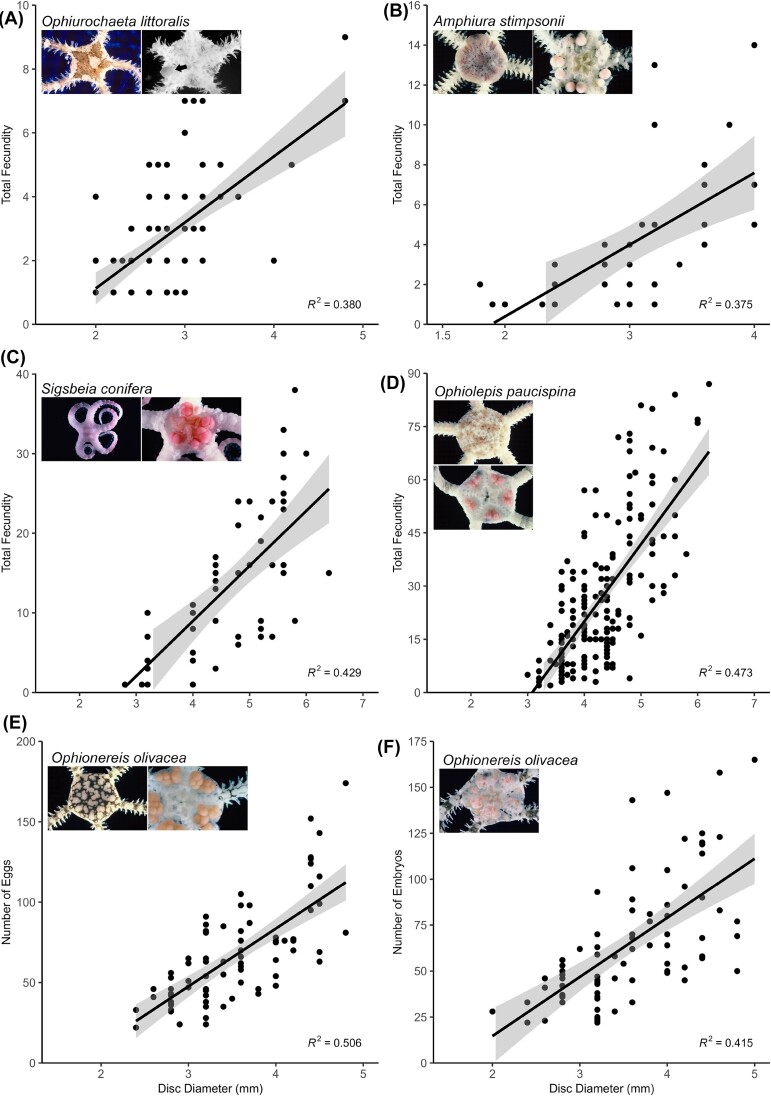
Relationship between disk diameter and fecundity in five Caribbean ophiuroids that care for their young. Images show the intact individual and dissected discs to show eggs or juveniles. Four of the species (A–D) have continuous reproduction with advanced eggs and young present year-round. (E–F) *Ophionereis olivacea* has seasonal reproduction with eggs and young present at different times of the year (data reanalysis from Byrne 1991). Shaded line indicates 95% confidence intervals. In the images of the dissected specimens, the juveniles were removed from the bursae to show the ovaries and eggs clearly. The black arrow in (A) points to the single egg. Disc diameters: A = 4.5 mm; B = 4 mm; C = 6 mm; D = 6 mm; E, F = 5 mm.

Thus far, all ophiuroids known to care for their young are hermaphroditic (Mortensen 1920; Hendler 1991), often with ovaries and testes on the same genital plates (Fig. 2G). Most of these species also have large eggs and are ovoviviparous developers (Fig. 2E, F, Fig. 3, Supplementary Table S1).

For the viviparous species where the egg has been secondarily reduced and the young are provisioned with extra-embryonic nutrition, the small eggs contrast with the large juveniles that emerge from the bursae (Supplementary Table S2). This is shown for *Amphipholis squamata*, which has a small egg (∼ 100 µm diameter) and gives rise to large juveniles (1200 µm maximum disc diameter, 13 arm segments, Hendler 1991). (Fig. 2B, C). For *A. squamata*, extraembryonic nutrients are provided by the hemal sinus (Fig. 2D). As shown in a comparison of gonads of *A. squamata* and that of its congener with planktotrophic development, *A. pugetana*, the switch to viviparity is associated with marked reduction in egg production with just one egg evident in the ovary of *A. squamata* (compare Fig. 2A, C).

In histological sections, the eggs of the ovoviviparous species had a homogeneous PAS+ response indicating dense deposition of yolk protein and less lipid provisioning (Fig. 2E, F). These eggs were negatively buoyant when teased out of the ovary. The intense PAS+ reaction is also evident in the stomach of the developing juveniles (Fig. 2E), showing the storage of maternal nutrients in the cells of the digestive system to support development. This is also evident in the color of the gut of live juveniles (Fig. 2H). These ophiuroids incubate their young to an advanced stage, emerging from the bursa with 2–12 arm segments (Fig. 2I) (Byrne 1991; Hendler 1991).

Four of the ovoviviparous species (*Amphiura stimpsoni, Ophiolepis paucispina, Ophiurochaeta littoralis*, and *Sigsbeia conifera*) are simultaneous hermaphrodites (Fig. 2G). *Ophionereis olivacea* is a protandric hermaphrodite (Fig. 3E) with smaller individuals being male and larger individuals being female or hermaphroditic (Byrne 1991). The eggs of the ovoviviparous species are very large (largest *Sigsbeia conifera* 760 µm diameter, see Supplementary Table S1) and so are easily counted in dissected ovaries (Fig. 3A-E). These ophiuroids have low fecundity with mean total fecundity ranging from 28–47 eggs/embryos (*Amphiura stimpsonii, Ophiolepis paucispina, Ophiurochaeta littoralis, O. olivacea*, and *Sigsbeia conifera*) (Fig. 3). These species had progeny in the gonads at all sampling times (see methods). The fecundity assessment in Fig. 3 may be underestimated on an annual basis because the data for some species were collected only at a few time points. The data show the low number of eggs produced by ovoviviparous species that care for their young (Fig. 3), compared with species that have planktonic larvae (Fig. 1A, B, E, F; Fig. 2A). There was a significant positive relationship (*P* < 0.001) between fecundity/clutch size and adult size in all five species (Fig. 3).

### Egg size distribution with respect to development mode

**Fig. 4:**
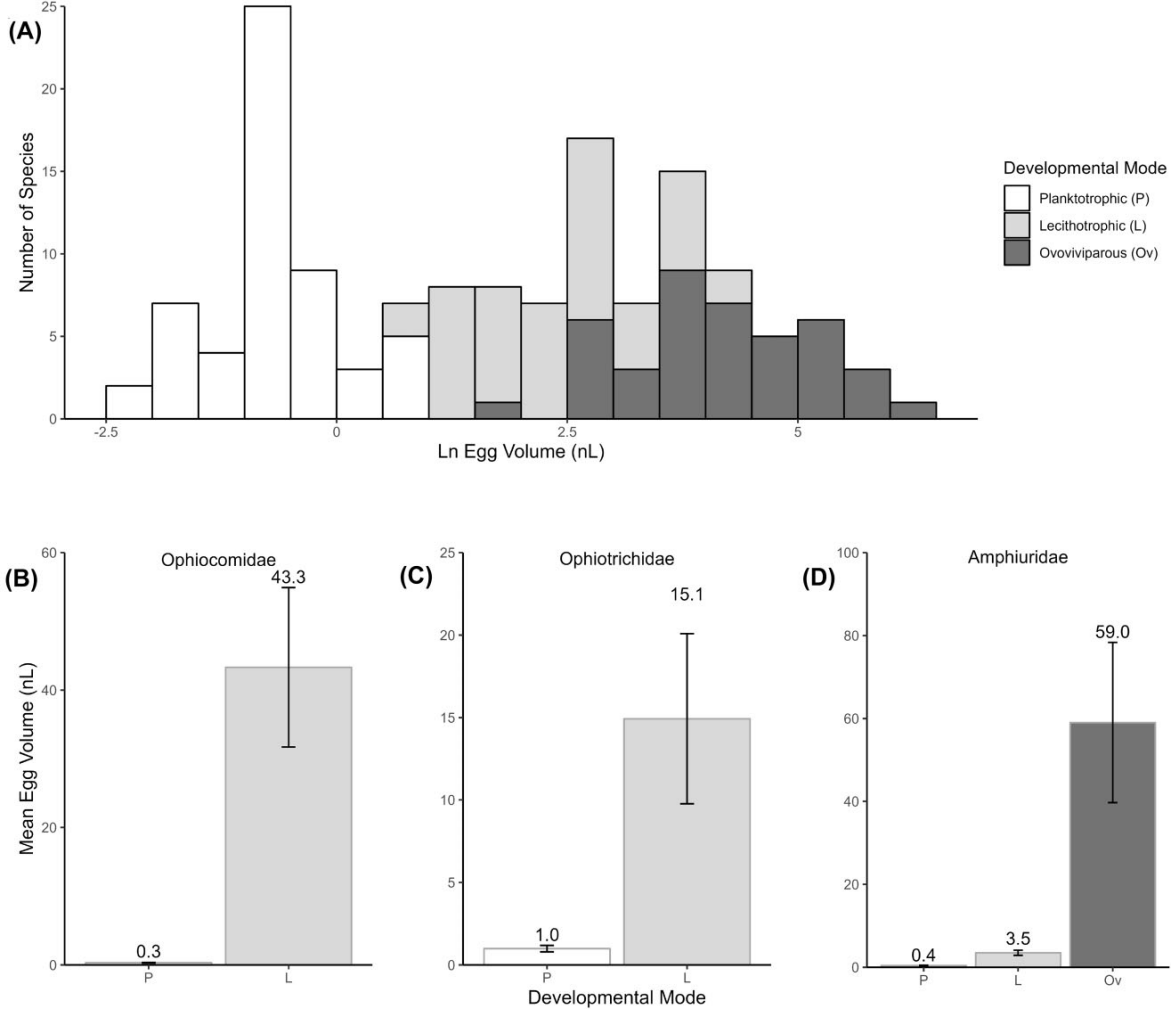
The distributions of egg volume and developmental mode in ophiuroids. (A) total data set (*n* = 140). (B) Ophiocomids, mean egg volume (SE) (planktotrophs, *n* = 17; lecithotrophs, *n* = 5). (C) Ophiotrichids, mean egg volume (SE) (planktotrophs, *n* = 12; lecithotrophs, *n* = 4). (D) Amphiurids, mean egg volume (SE) (planktotrophs, *n* = 5; lecithotrophs, *n* = 7; ovoviviparous species, *n* = 7).

**Fig. 5:**
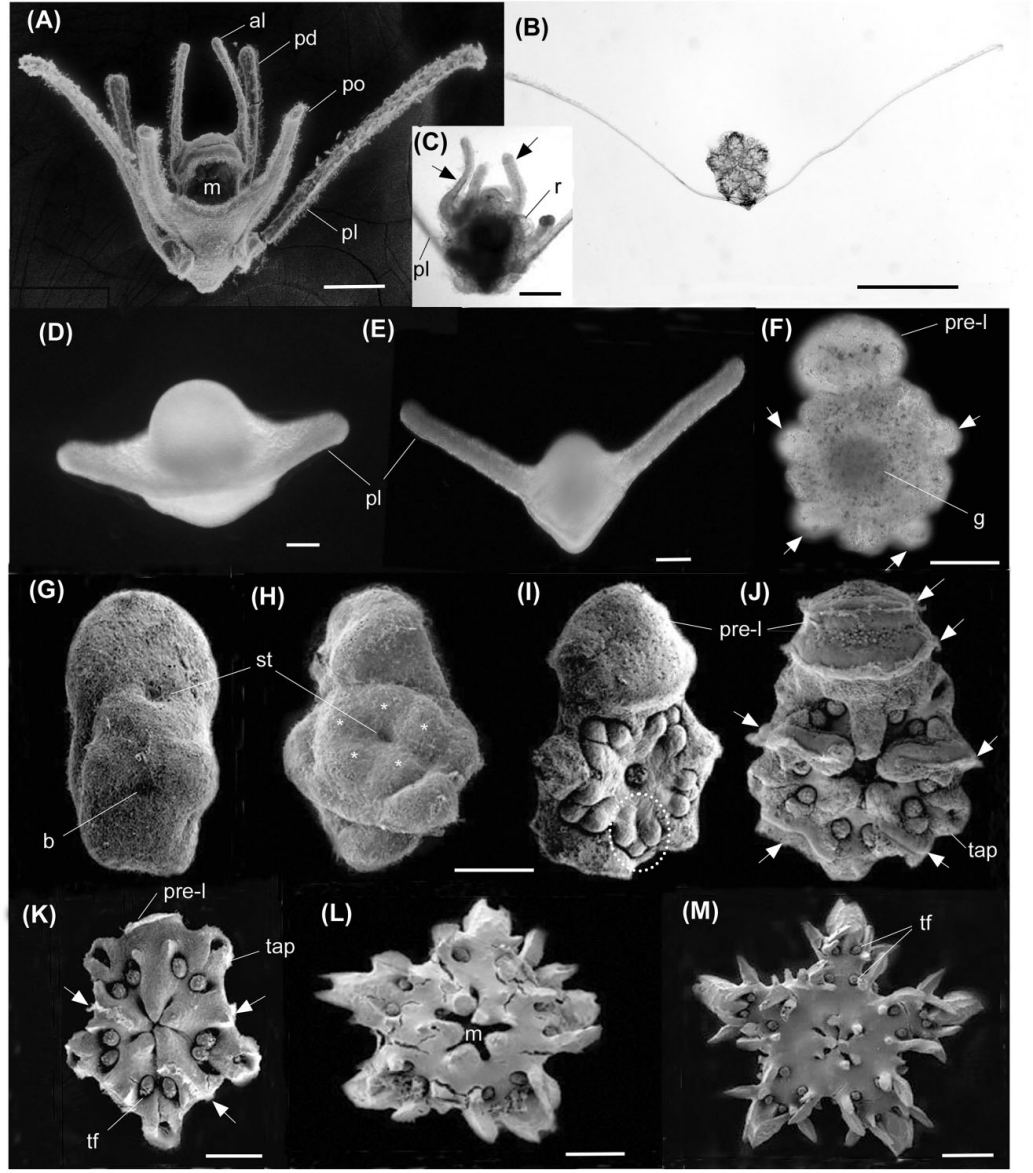
Light and scanning electron micrographs of ophiuroid larvae, metamorphosis, and juveniles. (A–C) Ophiopluteus of *O phiactis resiliens* (A) Eight-armed ophiopluteus with classic Type I development. Metamorphosis occurs in the plankton and involves resorption of the inner larval arms (C, arrows) as the juvenile is supported by the long posterolateral arms (illustrated in B), which are later discarded. (D–E) Yolky opaque lecithotrophic ophiopluteus of *Macrophiothrix nereidina*. (F) Vitellaria of *C larkcoma pulchra*. The round sphere where the juvenile gut (JG) is developing includes the remaining egg reserves. The arrows point to ciliary tracts. (G) Early vitellaria of *Ophiarachnella ramsayi*. The blastopore (B) is not yet closed. (H–M) Vitellaria development to the juvenile in *Ophioderma cinereum*. The developing juvenile is first evident as five bulges of the five radii (H, stars) around the stomodeum (ST). Developing tube feet (TF) appear in each radius (circled in I). Arrows show ciliary tracts. (K–M) Early juveniles of *O. cinereum* with settlement on day 4 (K) with the remains of the pre-oral lobe (PRE-L) and ciliary tracts present (arrows) and older 10 day (L) and 14 day (M) old juveniles, the latter with the mouth open and has two pairs of tube feet (TF) in each radius. The terminal tube foot is withdrawn into the pore of the terminal arm plate (TAP). al, anterolateral arms; m, mouth; pd, posterodorsal arms; pl, posterolateral arms; po, postoral arms. Scale bars: A, C–M = 100 µm; b = 500 µm. Note for G–J scale in row of images applies to all. (D–E, courtesy Dr Jonathan Allen, College of William and Mary).

Egg size data were available for 140 species (Supplementary Table S1), 103 with planktonic development through feeding or non-feeding larvae and 39 ovoviviparous species that care for their young (Fig. 4, Supplementary Table S1). For species where planktonic larvae have been reared, 46 have planktotrophic ophioplutei, 6 have lecithotrophic ophioplutei, and 27 species have lecithotrophic vitellaria larvae (Figs. 4 and 5, Supplementary Table S1). Based on measurements of eggs in dissected ovaries from this and previous studies, nine species were designated to have planktotrophic larvae, and fourteen to have lecithotrophic larvae (mode of development indicated in parentheses in Supplementary Table S1).

The ln-transformed egg volume data show two main groups: one corresponding to planktotrophic larvae and the other a combination of lecithotrophic larvae and ovoviviparous parental care, with the latter being on the right side of the distribution (Fig. 4). Gaussian mixture modeling revealed that the egg volume data were bimodal (Gaussian mode means: -0.872, 2.918; component variances: 0.402, 2.173). A log-likelihood ratio test comparing the optimized bimodal model with a unimodal model showed that the egg volume data were statistically unlikely to have a unimodal distribution (*P* < 0.001). The two species with a facultatively feeding larva, which can use exogenous food but do not need to feed to achieve the juvenile stage, [*Amphiodia* species “opaque”—see Nakata and Emlet (2023) and *Macrophothrix rhabdota*, Supplementary Table S2] have Ln egg volume values of 0.36 and 1.85, respectively. When added to the egg-size distribution, the eggs of these species are positioned intermediate between the two planktotrophy-lecithotrophy modes (Supplementary Fig. S1).

Analysis of the egg volume data indicated that egg size differed across the three life history types (ANOVA: Welch’s F_(2,80.1)_ = 369.11; *P* < 0.0001; Games-Howell post hoc test: planktotrophy < lecithotropy < ovoviviparity). The mean egg volume of species with planktotrophic larvae was 0.69 nL (SE = 0.087, range 0.087–3.05 nL, *n* = 55). The mean egg volume of species with lecithotrophic larvae was 15.59 nL (SE = 2.3 nL, range 1.77–87.1 nL, *n* = 46). For the ovoviviparous species, mean egg volume was 108.89 nL (SE = 18.39, range 6.37–523.59 nL, *n* = 39).

For ophiuroid families that include planktotrophic and lecithotrophic species’ development with representation of >10 species, (Ophiocomidae, Ophiotrichidae, and Amphiuridae), the dichotomy of egg size is apparent (Fig. 4B-D). For the Ophiocomidae and Ophiotrichidae, species with feeding and non-feeding larvae had mean egg volumes of 0.3 and 43.3 nL; and 1.0 and 15.1 nL, respectively (Fig. 4B, C). For the Amphiuridae species with feeding, non-feeding larvae, and ovoviviparous development, the mean egg volumes were 0.4, 3.5, and 59 nL, respectively (Fig. 4D). Some genera or families are dominated by lecithotrophic development. *Ophionereis* is known to have just one species with a feeding ophiopluteus and seven species with non-feeding vitellaria (Supplementary Table S1). The Ophiodermatidae is the only ophiuroid family known to have lecithotrophic development through a vitellaria larvae as the single larval stage.

### Larval patterns—Type I vs Type II development, larval form at metamorphosis

**Fig. 6:**
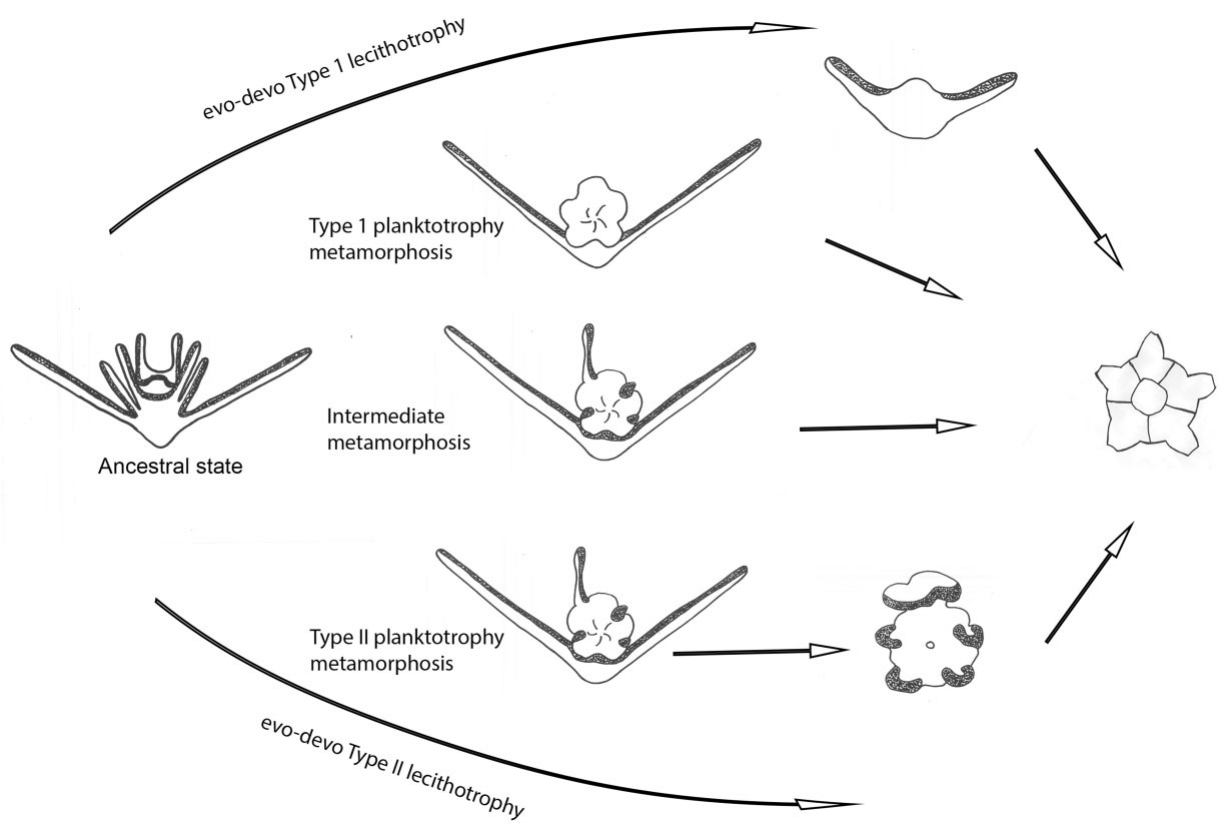
Hypothetical pathways in evolution of development (evo-devo), metamorphic phenotypes in ophiuroids. Type I development, metamorphosis via a developing juvenile supported by the two posterolateral arms. Evolution of Type I lecithotrophy involves a yolky reduced ophiopluteus. Type II development, metamorphosis via transformation to a non-feeding vitellaria. Intermediate (Type I/II) metamorphosis via a developing juvenile supported by the two posterolateral arms, but also retaining the right anterolateral arm and inter-radial ciliary ridges from resorbed larval arms. Type II lecithotrophic development in species that just have a vitellaria larva. From Selvalumaraswany and Byrne (2023). See Fell (1948) for an illustration of the progressive reduction in the number of ophiopluteal arms with receding time to metamorphosis.

Metamorphosis in ophiuroids can appear strikingly different depending on the larval form that supports the developing juvenile (Figs. 5 and 6). For species with planktotrophic development, in the Type I pattern, metamorphosis occurs in the plankton as the juvenile is supported by the last remaining ophiopluteus arms (posterolaterals). As shown for *O. resiliens*, the ophiopluteus is the metamorphic larva, and the posterolateral arms are discarded (Fig. 5A-C). In other species, final loss (discarded or resorbed) of the posterolaterals at metamorphosis varies. Some ophioplutei show partial or fleeting vitellaria-like traits, features intermediate between Type I and II development (Fig. 6).

For species with lecithotrophic development, the larval type at metamorphosis can be a yolky reduced ophiopluteus in the Type I pattern, as illustrated for *Macrophiothrix nereidina*. This is known for six species (Fig. 5D, E, Fig. 6, Supplementary Table S1). The pattern in reduction in the number of arms that ophioplutei develop with decreasing in time to metamorphosis, is illustrated by Fell (1948). The most extreme case known is for the yolky 2-armed ophiopluteus of *M. nereidina* (Allen and Podolsky 2007).

In the Type II pattern, in species with a planktotrophic development, the ophiopluteus transforms into the armless vitellaria larva at metamorphosis through resorption of the larval arms and rearrangement of the ciliary band into ciliary tracts. This is only known for the Ophiocomidae (see Byrne et al. 2024). The vitellaria settles by attaching with the juvenile tube feet, followed by completion of metamorphosis on the benthos. Other ophiuroids with lecithotrophic development have only the vitellaria, the Type II pattern, as shown for *Clarkcoma, Ophiarachnella*, and *Ophioderma* species (Fig. 5F–M).

## Discussion

Life history diversity in the Ophiuroidea, the most speciose echinoderm class, is extensive with multiple developmental modes (planktotrophic larvae, facultative planktotrophy, lecithotrophic larvae, oviviviparity, viviparity), larval developmental patterns (Types I and II), and the location of development (planktonic, benthic). This developmental diversity, including among closely related species, presents an opportunity to use the Ophiuroidea as a model system for evo-devo research. Many studies have overcome the challenge of spawning and rearing ophiuroids through metamorphosis (see Hodin et al. 2019). These advances, together with the phylogeny now available for the Ophiuroidea and revised taxonomy (O'Hara et al. 2017, 2018), are key to understanding evo-devo in this class.

We revisited the question as to whether the size distribution of ophiuroid eggs is unimodal or bimodal. In asteroids and echinoids, there is a bimodal distribution in Ln egg diameter reflecting planktotrophic and lecithotrophic development (Sewell and Young 1997). The dataset used in Sewell and Young (1997) for the ophiuroids was dominated with species with unknown development. Here we used a significantly larger ophiuroid egg size dataset, where developmental mode was known, and used egg volume as the parameter (see Moran and McAlister 2009). For species that care for their young, we used only data from ovoviviparous species to avoid the confounding influence of extra-embryonic nutrition. We also did not include the two species with development through facultative planktotrophy but illustrated their position in the egg size distribution (Supplementary Fig. S1). We found that egg size distribution in ophiuroids had two Gaussian modes. The eggs of the ovoviviparous species were the largest observed. As a result, there were three statistically significant egg size groupings: planktotrophy, planktonic lecithotrophy, and ovoviviparity.

The egg sizes of the two species with a facultatively feeding larva are intermediate between the planktotrophy-lecithotrophy egg size Gaussian modes (Supplementary Table S2; Supplementary Fig. S1). Interestingly, *Macrophothrix caenosa*, with eggs just slightly larger (242 µm diameter, 7.42 nL volume) than those of the facultative planktotroph (*M. rhabdota*; 230 µm diameter, 6.37 nL volume), has a lecithotrophic ophiopluteus with a full array of larval arms and a digestive tract that appears to be functional, but despite this does not feed (Allen and Podolsky 2007). The rarity of echinoderms (e.g., two ophiuroid species) with a facultatively feeding larva supports the hypothesis that facultative planktotrophy is an evolutionarily unstable strategy with selection favoring planktotrophy or lecithotrophy (Vance 1973; Hart 1996; Wray 1996). There also appears to have been selection to provide excess energy in species with large eggs to produce a larger, likely more resilient juvenile (Emlet and Hoegh-Guldberg 1997; Allen and Pernet 2007; Byrne and Sewell 2019), as seen in ophiuroids with non-feeding larvae and ovoviviparity.

While ophiuroid egg-size trends reflect those seen in other echinoderms, a few differences are evident. Ophiuroids with planktotrophic or lecithotrophic larvae often have eggs that are smaller compared with other echinoderms with these modes of development (Falkner et al. 2015). For the species with planktotrophic larvae, many ophiuroids have eggs in the size range 50–80 µm diameter, among the smallest known for echinoderms with this type of larva. Species with similarly sized eggs are known only for a few echinoids and synaptid holothuroids (McEdward and Miner 2001). *Amphiura chiajei* has the smallest egg (1.77 nL volume, 150 µm diameter) known for an echinoderm with non-feeding larvae (Fenaux 1963; Hendler 1975; McEdward and Miner 2001), and other amphiurids with lecithotrophic development also have small eggs (Supplementary Table S1). Energetic provisions in such comparatively small eggs for species that have non-feeding larvae are suggested to approach the lower level required to support lecithotrophic development to the juvenile (Falkner et al. 2006). Ophiuroids may provision their eggs differently to other echinoderm classes because there is a greater variety of the types of energetic lipids in the eggs of lecithotrophic species compared with this mode of development in the other classes (Falkner et al. 2015).

Differences in feeding rates and metabolism between ophiuroid and echinoid plutei in species with similar egg size, and reared at the same temperature, point to differences in development (Hart 1996; Whitehill and Moran 2012). Ophioplutei have lower specific clearance rates, fewer cilia per length of ciliated band, lower water current velocities, poorer feeding capabilities, slower development, and lower metabolism compared with echinoplutei (Hart 1996; Whitehill and Moran 2012). Their lower metabolic rate may be linked to their lower food clearance rates (Hart 1996). Thus, the trend for smaller egg sizes in some ophiuroids with planktotrophic development may have a physiological basis. As more data emerge on the size and biochemical content of echinoderm eggs, we will obtain a better understanding of egg evolution to see how the details differ within and between echinoderm families and classes.

The eggs of echinoderms with non-feeding larvae typically contain more energetic reserves than needed to achieve the juvenile stage, indicating selection to produce larger and better provisioned juveniles (Turner and Rutherford 1976; Emlet and Hoegh-Guldberg 1997; Allen and Pernet 2007; Byrne and Sewell 2019). Extreme examples of this are seen for the ovoviviparous ophiuroids, where offspring emerge as very large juveniles with 7–12 arm segments. The allocation of maternal provisions to the juveniles was clearly seen in the strong egg pigments in the developing gut of the live juveniles of *Sigsbeia conifera* and *O. olivacea* and in histological sections of *Ophiolepis paucispina*. The exception is *Ophiopeza spinosa*, which has the smallest egg (300 µm diameter) known for the ovoviviparous ophiuroids, releasing small juveniles with just 2–3 arm segments (Byrne et al. 2008).

The strong PAS+ response in histological sections of the eggs of the ovoviviparous species indicates the dominance of yolk protein. This is likely associated with selection for egg biochemical constituents to promote negative buoyancy to maintain the large eggs of these species in the benthos, as seen for the benthic eggs of asteroids (Chia 1968; Turner and Lawrence 1979; Byrne 1992; Byrne et al. 2003). The eggs of asteroids that have benthic development in species that care for their young (e.g., *Leptasterias hexactis*) or lay benthic egg masses (e.g., *Parvulastra exigua*) have intensely PAS+ eggs with little to no evidence of lipid droplets at the histological level (Chia 1968; Byrne 1992). Egg biochemistry revealed that the eggs of *Parvulastra exigua* have a protein/lipid ratio similar to that for the eggs of species with planktotrophic development (Prowse et al. 2008). These observations for ophiuroids and asteroids indicate hypertrophy in vitellogenesis (yolk protein production) during egg development and a potential reduction in lipid production. For ophiuroids, this hypothesis warrants testing through comparative biochemical analyses of maternal provisioning in closely related species that have small and large eggs and planktonic and benthic development, respectively.

As shown for *Amphipholis squamata*, viviparous ophiuroids also give rise to very large juveniles, indicating extensive extraembryonic nutrition with the maternal hemal system as the likely source of nutrients (Fell 1946; Walker and Lesser 1989; Byrne 1994). In addition to being viviparous, this species is unusual in having high levels of inbreeding or clonality and likely to have asexual reproduction, despite the presence of male and female gametes (Hugall et al. 2023). For *Ophionotus hexactis*, the intragonadal juveniles exhibit a 2200-fold increase in dry mass compared with that of the egg and may involve utilization of nurse eggs or parental body fluid (Turner and Dearborn 1979).

The trade-off between fecundity and the amount of energy invested in eggs is exemplified by the ovoviviparous species. Most ophiuroids that care for their young release a few eggs at a time and have small clutches. Although the annual fecundity could not be determined for the species with asynchronous reproduction, the number of offspring produced on an annual basis likely reflects that determined here, as individual offspring may reside in the bursa for some time. Ophiuroids with planktonic larvae, by contrast, have a much higher fecundity producing 10’s to 100’s of thousands of eggs (Rumrill and Pearse 1985; Hendler 1991; Selvakumaraswamy and Byrne 2000b). The limitation on offspring production in viviparous and ovoviviparous ophiuroids is likely due to allometric, spatial constraints to accommodate young in the bursal space (Strathmann and Strathmann 1982; Byrne 1991). The offspring are typically crowded in bulging bursae. Ophiuroid bursae are densely ciliated, thin-walled invaginations used for respiration and are likely to be preadaptive features for evolution of parental care through retention of the eggs in a structure ideal for protection of embryos (Hendler 1979). Other less common forms of parental care include offspring development in the gonads (e.g., *Ophionotus hexactis*) and externally on the oral surface (e.g., *Ophiothrix synoecina*) (Turner and Dearborn 1979; Hendler 1991; Schoppe and Hol 1994; Schoppe 1996).

Parental care of offspring is a remarkable trait of the Ophiuroidea and has long attracted attention (Mortensen 1920; Hendler 1977, 1991), as it appears to be more prevalent in ophiuroid genera than in other echinoderms (see McEdward and Miner 2001). This mode of reproduction has independently evolved across many ophiuroid families with contrasting support strategies: from ovoviviparity, in providing nutrients in energy-rich eggs, to viviparity, in having secondarily reduced eggs, and a switch to matrotrophy. There is also a strong link between small adult size, hermaphroditism, and parental care in ophiuroids (Mortensen 1920; Hendler and Littman 1986; Byrne 1991; Hendler 1991), as is common in marine invertebrates (Chia 1974; Srathmann and Strathmann 1982; Strathmann et al. 1984; Sewell 1994). Parental care in ophiuroids appears to be linked to paedomorphic change (Hendler 1979, 1991). This is evidenced by very early maturation in *O. olivacea* with testes appearing in tiny individuals (1.5 mm DD) shortly after they vacate the bursa (Byrne 1991). Exceptions to this small size trend include *Ophioplocus esmarki, Ophionotus hexacitis*, and *Ophioderma wahlbergii* (disc diameter 18, 25, and 30 mm, respectively) (Turner and Dearborn 1979; Rumrill and Pearse 1985; Landschoff and Griffiths 2015).

The presence of functional (*Ophiopeza spinosa*) or reduced (*O. esmarki, O. olivacea*) vitellaria larvae in the bursae, provides evidence that evolution of parental care involved an ancestral form that had a planktonic vitellaria. In contrast, parental care in *Ophionotus hexactis* and *Amphipholis squamata* includes a vestigial ophiopluteus stage, indicating evolution through an ophiopluteal pathway (Mortensen 1921; Fell 1946). The presence of a functional vitellaria in the bursae of *Ophiopeza spinosa* that can develop to the juvenile independently of the parent shows that this species has potential to brood and broadcast its larvae (Byrne et al. 2008). Facultative parental care may occur in ophiuroids in fragmented deep-sea habitats (e.g., seamounts) (see O'Hara et al. 2014).

The long-standing paradigm known as “Thorson’s Rule” that posits, in response to cold, food limited conditions, the number of marine invertebrate species with pelagic feeding larvae (and thereby small eggs) should decrease with increasing latitude and in deeper water with an increase in non-pelagic development (Thorson 1950) has generated much thought and discussion (Pearse et al. 1991; Pearse 1994; Pearse and Lockhart 2004; Marshall et al. 2012). For ophiuroids, a meta-analysis found that there was no relationship between latitude and egg size (Marshall et al. 2012). Similarly, there is no latitudinal or depth trend in non-pelagic development in echinoderms (Pearse 1994). Rather than latitudinal or depth gradients, trends in life history modes appear to be more influenced by vicariant histories and speciation within clades (Pearse 1994; Pearse and Lockhart 2004), as seems the case for ophiuroids. For instance, lecithotrophic larvae are prevalent in some tropical ophiuroid taxa (e.g., Ophiodermatidae, *Ophiomastix* spp.), while in the tropical *Ophiocoma* spp., planktotrophic larvae dominate. Interestingly, there are biogeographic trends with large egg-ovoviviparity common in ophiuroids from the tropical Atlantic, but this mode of reproduction is rare in species from the tropical Indo-Pacific (Mortensen 1920; Hendler 1975, 1991; Byrne 1991; Byrne et al. 2008). Parental care and maximum egg size may also be related with selection to remain small and reproduce at an early age (paedomorphosis), as seen in ctenophores that reproduce at a minute body size and early in their ontogeny (Edgar et al. 2022). For ovoviviparous ophiuroids, diminutive size may also be related to habitat in association with life in a chemically protected habitat (Hendler 1979; Byrne 1991). Many ophiuroids that care for their young live in microhabitats in the interstices of beds of the calcareous alga *Halimeda* (Byrne 1991), which is known to produce chemicals that deter grazing by fishes (Paul and Fenical 1983). Interpreting biogeographic trends in larval type and extent of maternal investment in ophiuroids at a global scale, and the potential selective factors involved, are best conducted within a robust phylogenetic framework and in consideration with ecological factors.

Ophiuroid larval form has a strong influence on swimming and dispersal. A suspended juvenile carried in the plankton by two long larval arms (Type I) may have enhanced swimming speed and weight-bearing capacity with stability in shear aided by the angle of the arms (Grunbaum and Strathmann 2003; Strathmann 2020). These larvae would be expected to have greater dispersal capacity than larvae that resorb these arms (Type II). This is also indicated by the presence of juveniles in plankton samples (Hendler et al. 1999). In some species (e.g., *Ophiopholis aculeata*), the discarded arms of Type I ophioplutei have the potential to regenerate a completely new larva as a clonal mode of asexual reproduction (Balser 1998). Larval clonality may also have influenced selection to maintain Type I development in some clades to facilitate dispersal and asexual reproduction in the open ocean. That said, most assessments of marine invertebrate planktonic larval dispersal are based on shallow-water species (e.g., Shanks 2009). In the deep-sea, where non-feeding larvae are prevalent, including for ophiuroids, the suggestion is that due to very low metabolic rates in very cold water, and associated long pelagic larval development time, lecithotrophy increases dispersal distance (Young et al. 1997; Young 2003). The presence of transverse ciliary bands in ophiuroid vitellariae may also confer greater swimming speeds (Strathmann 2020).

The distribution and expression of Type I and Type II development vary across ophiuroid families. For species with planktotrophic development, the metamorphic larva differs among families with the Type I pattern, as shown here for *O. resilien*, is characteristic of the Ophiotrichidae, Ophiactidae, Ophiopholidae, and Amphiuridae families all in the suborder Gnathophiura (O'Hara et al. 2017; Selvakumaraswamy and Byrne 2006, 2023). The Type II pattern where the ophiopluteus transforms into a vitellaria is seen thus far only in the Ophiocomidae (Mladenov 1985; Byrne et al. 2024). Different independent evolutionary paths to lecithotrophy are also evident. The Amphiuridae and Ophiotrichidae have non-feeding ophioplutei (Type I) (Fenaux 1963, 1970; Mladenov 1979; Allen and Podolsky 2007), whereas at least four ophiuroid families (Hemieuryalidae, Ophiodermatidae, Ophiolepididae, and Ophoipezidae) have development through a non-feeding vitellaria (Type II) only (Hendler 1991; Cisternas and Byrne 2005; Byrne et al. 2024). Given that non-feeding yolky ophiopluteus and vitellaria larvae are present in species with similarly sized eggs (e.g., *Macrophiothrix nereidina* and *Ophionereis schayeri*) (Selvakumaraswamy and Byrne 2000b; Allen and Podolsky 2007), it appears that egg size alone does not correlate with the larval pattern (Type I, Type II).

With respect to the question as to whether the Type I or Type II larval pattern is ancestral for the Ophiuroidea (Mladenov 1985; Hendler 1991; McEdward and Miner 2001), the answer is not clear. The presence of vestigial ophiopluteal features (incomplete gut and larval skeleton) in some vitellaria larvae provides a link to an ophiopluteal ancestral form (Hendler 1982; Mladenov 1985; Selvakumaraswamy and Byrne 2004). In addition, with detailed examination, the ciliary tracts of vitellaria larvae can be linked to ophiopluteal ciliary bands (Fourgon et al. 2005). Fourgon et al. (2005) pointed out that the main substantive difference between the two types of development is the persistence of the posterolateral arms, which are discarded (Type I) or which are resorbed (Type II). That said, in *Ophiothrix ciliaris*, a species with Type I development, the posterolateral arms are resorbed, but there is no vitellaria (Selvakumaraswamy and Byrne 2006). McEdward and Miner (2001) suggesting that the ancestral pattern of ophiuroid development involved an ophiopluteus larva that transforms into a vitellaria larva before metamorphosis (Type II). Mladenov (1985) suggested that Type I development arose through loss of the vitellaria.

The presence of transformation to a second larva with transverse ciliary bands (doliolaria) in sea cucumbers with planktotrophic (auricularia) larvae and the presence of the vitellaria-like larvae in crinoids (Lacalli and West 2000; Nakano et al. 2003) could be taken as potential outgroup evidence that Type II development is ancestral for ophiuroids. However, the presence of a barrel-shaped larva with cross-body ciliary bands in many echinoderm families is also suggested to indicate functional requirements (Lacalli 1993; Lacalli and West 2000) and likely to be a convergent morphology (McEdward and Miner 2001).

The fleeting vitellaria features seen in some ophioplutei at metamorphosis (even variable within clutches of larvae) based on the pattern of larval arm resorption and hints of ciliary tract formation (e.g., *Ophionereis, Ophiodaphne, Ophiothrix, Ophiactis*), show the marked morphological plasticity at metamorphosis in some species (Byrne and Selvakumaraswamy 2002; Tominga et al. 2004; Selvakumaraswamy and Byrne 2006, 2023). This variation has been taken to indicate an ancestral morphological plasticity at metamorphosis followed by canalization in some taxa to the vitellaria metamorphic phenotype (Fourgon et al. 2005; Selvakumaraswamy and Byrne 2023). In this scenario, Type I development appears to be ancestral with evolution of the vitellaria associated with retention of the ophiopluteal ciliary bands expressed as ciliary tracts.

The range of metamorphic phenotypes from the ophiopluteus with prominent posterolateral arms through metamorphosis and then discarded to intermediate patterns of gradual arm resorption sometimes resulting in fleeting vitellaria features to fully developed vitellariae indicates that the Types I and Type II patterns are likely to be two ends of a morphological continuum (Cisternas et al. 2004; Selvakumaraswamy and Byrne 2023). To understand the ancestral pattern for the Ophiuroidea (Type I or Type II), more data on development through metamorphosis are needed for species with ophioplutei with larval characters mapped to the phylogeny for this echinoderm class (O'Hara et al. 2017).

With regard to climate change and prospects for ophiuroids, species responses will be influenced by developmental mode. Life history, biology, and ecology point to potentially stress-resilient species. Ocean warming and acidification appear to be particularly deleterious for larvae that need to feed and which also need to calcify (Byrne 2011; Prezeslawski et al. 2015). Thus, ophiuroids with ophiopluteal development may be particularly vulnerable. Species with lecithotrophic larvae and ovoviviparous species, where egg reserves are set aside for the juvenile, may be energetically buffered from climate-related stress. As found for oyster young in the brood space (Gray et al. 2019, 2022), ophiuroid young in the bursae likely experience acidified conditions due to buildup of respiratory CO_2_. While this can be deleterious, it is suggested that development under these conditions may provide a physiological adaptation to high CO_2_ and resilience to ocean acidification (Gray et al. 2019, 2022). Parental care benefits offspring by protecting them from adverse environmental conditions and ensures that offspring are released into suitable habitat as large progeny likely with enhanced mobility and less vulnerability to predation (Hendler 1975, 1991), albeit with the reproductive trade-off of low clutch size. Ophiuroids with non-planktonic development in caring for their young may have the greatest potential to be resilient in the face of environmental change. While there is little evidence of selective extinction of species with feeding larvae in the fossil record (Nützel 2014; Strathmann 2020), given the fast pace of contemporary climate change, it seems that ophiuroids with larvae that have to calcify (e.g., ophioplutei) may be the most vulnerable to climate change. Development through a non-feeding, non-calcifying larva (e.g., vitellaria) may provide some resilience.

## Supplementary Material

icae048_Supplemental_Files Figure S1. The distributions of egg volume and developmental mode in ophiuroids with egg sizes of the facultative planktotrophy included (see legend Fig. 4). Egg size in the two species with facultative planktotrophy, *Amphiodia* sp. (opaque) and *Macrophiothrix rabdota* (see Allen and Podolsky 2007; Nakata and Emlet 2023) have intermediate positions in the overall egg size distribution.

## Data Availability

All data are available within the manuscript and supplementary material.
