# Supplementary material for: Evolution of Maternal Provisioning and Development in the Ophiuroidea: Egg Size, Larval Form, and Parental Care
Source: Integr Comp Biol. 2024 May 23;64(6):1536–55. doi: 10.1093/icb/icae048 (PMC11659680; doi:10.1093/icb/icae048)
Supplement: icae048_Supplemental_Files — Figure S1. The distributions of egg volume and developmental mode in ophiuroids with egg sizes of the facultative planktotrophy included (see legend Fig. 4). Egg size in the two species with facultative planktotrophy, Amphiodia sp. (opaque) and Macrophiothrix rabdota (see Allen and Podolsky 2007; Nakata and Emlet 2023) have intermediate positions in the overall egg size distribution. [file icae048_supplemental_files.zip › icb-2024-0007-File008.docx]

**Supplementary Table 1**. Available data for ophiuroid egg size, mode of development (Dev) and larval type (excluding matrotrophic species, see methods, Table S2). Data from the literature were sourced from previous reviews with check of source (Hendler et al. 1995: McEdward and Miner 2001), and more recent publications (as indicated). Abbreviations: BE, benthic embryos/larva/young; YE, young external, under/attached to parent’s body; L, lecithotrophic development; O, ophiopluteus; O(NF), non-feeding ophiopluteus; Ov, ovoviparous; P, planktotrophic development; Po, polar; RV, reduced vitellaria; Te, temperate; Tr, Tropical; V, vitellaria; -, no data; *, maximum egg diameter in dissected gravid ovaries, #* data not used for analysis; (P), (L) or (Ov), mode of development inferred from egg size.

| **Family & Species** | **Egg diam (µm)** | **Egg vol (nl)** | **Dev Mode** | **Larval type/ Benthic Dev.** | **Reg-ion** | **Reference** |
| --- | --- | --- | --- | --- | --- | --- |
| **Amphilepidida** |  |  |  |  |  |  |
| **Amphilipididae** |  |  |  |  |  |  |
| *Amphilepis ingolfiana* | 300 | 14.137 | (L) | - | Te | Schoener 1972 |
| **Amphiuridae** |  |  |  |  |  |  |
| *Amphiodia sp* | 100 | 0.524 | (P) | O | Te | Rumrill & Pearse 1985 |
| *Amphiodia akosmos* | 430 | 41.63 | Ov | BE | Te | Hendler & Bundrick 2001 |
| *Amphiodia occidentalis* | 190 | 3.59 | L | BE | Te | Emlet 2006 |
| *Amphiodia pulchella* | 65 | 0.144 | P | O | Tr | Hendler & Littman 1986 |
| *Amphiodia urtica* | 100 | 0.524 | P | O | Te | Emlet 2006 |
| *Amphioplus abditus* | 160 | 2.144 | L | BE | Te | Hendler 1975,1977 |
| *Amphioplus sepultus* | 180 | 3.054 | (L) | - | Tr | Hendler 1973, 1995 |
| *Amphipholis kochii* | 90 | 0.382 | P | O | Te | Yamashita 1985 |
| *Amphiura annulifera* | 300 | 14.137 | Ov | BE | Te | Mortensen 1924a |
| *Amphiura borealis* | 500 | 65.45 | Ov | BE | Te | Mortensen 1920a |
| *Amphiura capensis* | 300 | 14.137 | Ov | BE | Te | Mortensen 1920a |
| *Amphiura carchara* | 450 | 47.713 | Ov | BE | Te | Hendler & Tran 2001 |
| *Amphiura chiajei* | 150 | 1.767 | L | O(NF) | Te | Fenaux 1963, 1970 |
| *Amphiura filiformis* | 100 | 0.524 | P | O | Te | Mortensen 1920b, Bowmer 1982 |
| *Amphiura stimpsonii* | 680 | 164.636 | Ov | BE | Tr | Mortensen 1920a, 1921, Byrne 1991 |
| *Ophiocnida scabriuscula* | 210 | 4.849 | (L) | - | Tr | Hendler et al 1995 |
| *Ophiodaphne formata* | 90 | 0.382 | P | O | Tr | Tominaga et al 2004 |
| *Ophiophragmus filograneus* | 220 | 5.575 | (L) | - | Te/Tr | Stancyk 1970, 1973 |
| **Hemieuryalidae** |  |  |  |  |  |  |
| *Ophioplocus esmarki* | 330 | 18.817 | Ov | BE/RV | Te | Rumrill & Pearse 1985, Sweet et al. 2019 |
| *Ophioplocus hancoocki* | 150 | 1.767 | P | O | Te | Hendler 1991 |
| *Ophioplocus januarii* | 400 | 33.51 | (L) | - | Te | Brogger et al. 2013 |
| *Ophioplocus japonicus* | 300 | 14.137 | L | V | Te | Komatsu & Shoshaku 1993 |
| *Sigsbeia conifera* | 760 | 229.847 | Ov | BE | Tr | Hendler & Littman 1986, Byrne 1991 |
| **Ophiactidae** |  |  |  |  |  |  |
| *Hemipholis elongata* | 110 | 0.697 | P | O | Te | Heatwole & Stancyk 1982; Hendler et al 1995 |
| *Ophiactis quinqueradiata* | 65 | 0.144 | P | O | Tr | Hendler & Littman 1986 |
| *Ophiactis resiliens* | 83 | 0.299 | P | O | Te | Selvakumaraswamy & Byrne 2000 |
| *Ophiactis savignyi* | 100 | 0.524 | P | O | Tr | Hendler et al. 1985 |
| **Ophiolepididae** |  |  |  |  |  |  |
| *Ophiolepis cincta* | 300 | 14.137 | L | V | Tr | Mortensen 1938 |
| *Ophiolepis elegans* | 250 | 8.181 | L | V | TTe/Tr | Stancyk 1973 |
| *Ophiolepis impressa* | 200 | 4.189 | (L) | - | Tr | Hendler 1979a |
| *Ophiolepis kieri* | 350 | 22.449 | Ov | BE | Tr | Hendler 1979a |
| *Ophiolepis pacifica* | 200 | 4.189 | L | - | Tr | Hendler 1979b |
| *Ophiolepis paucispina* | 400 | 35.51 | Ov | BE | Tr | Byrne 1989, 1991 |
| **Ophionereididae** |  |  |  |  |  |  |
| *Ophionereis annulata* | 240 | 7.238 | L | V | Tr | Hendler 1982 |
| *Ophionereis fasciata* | 99 | 0.508 | P | O | Te | Selvakumarswamy & Byrne 2000 |
| *Ophionereis olivacea* | 400 | 33.51 | Ov | BE/RV | Tr | Byrne 1991 |
| *Ophionereis reticulata* | 250 | 8.181 | L | V | Tr | Hendler & Littman 1986 |
| *Ophionereis schayeri* | 240 | 7.238 | L | V | Te | Selvakumarswamy & Byrne 2004 |
| *Ophionereis squamulosa* | 200 | 4.189 | L | V | Tr | Mortensen 1921 |
| *Ophionereis vittata* | 200 | 4.189 | (L) | - | Tr | Hendler 1995 |
| *Ophionereis vivipara* | 230 | 6.371 | Ov | BE | Tr | Mortensen 1933a |
| **Ophiopholidae** |  |  |  |  |  |  |
| *Ophiopholis aculeata* | 120 | 0.905 | P | O | Te | Olsen 1942 |
| **Ophiopsilidae** |  |  |  |  |  |  |
| *Ophiopsila californica*#* | 220 | 5.575 | (L) | - | Te | This study |
| *Ophiopsila pantherina*#* | 382 | 29.187 | (L) | - | Tr | This study |
| *Ophiopsila riisei* | 230 | 6.371 | L | - | Te | Hendler & Littman 1986 |
| **Ophiothamnidae** |  |  |  |  |  |  |
| *Ophiopus articus* | 400 | 33.51 | (L) | BE | Po | Mortensen 1893; Thorson 1936 |
| **Ophiotrichidae** |  |  |  |  |  |  |
| *Macrophiothrix belli* | 406 | 35.041 | L | O(NF) | Tr | Allen & Podolski 2007 |
| *Macrophiothrix caenosa* | 242 | 7.421 | L | O(NF) | Tr | Allen & Podolski 2007 |
| *Macrophiothrix koehleri* | 147 | 1.663 | P | O | Tr | Allen & Podolski 2007 |
| *Macrophiothrix longipeda* | 155 | 1.95 | P | O | Tr | Allen & Podolski 2007 |
| *Macrophiothrix loridi* | 166 | 2.395 | P | O | Tr | Allen & Podolski 2007 |
| *Macrophiothrix nereidina* | 266 | 9.855 | L | O(NF) | Tr | Allen & Podolski 2007 |
| *Macrophiothrix spongicola* | 128 | 1.098 | P | O | Te | Selvakumaraswamy & Byrne 2006 |
| *Ophiothrix angulata*  *Ophiothrix caespitosa* | 100  100 | 0.524  0.524 | P  P | O  O | Tr  Te | Hendler et al. 1995  Selvakumaraswamy & Byrne 2006 |
| *Ophiothrix ciliaris* | 90 | 0.382 | P | O | Te | Selvakumaraswamy & Byrne 2006 |
| *Ophiothrix exigua* | 92 | 0.408 | P | O | Te | Kitazawa et al. 2014 |
| *Ophiothrix fragilis* | 100 | 3.054 | P | O | Te | McBride 1907; Smith 1940; |
| *Ophiothrix oerstedi* | 250 | 8.181 | L | O(NF) | Tr | Mladenov 1979 |
| *Ophiothrix savignyi* | 100 | 0.524 | P | O | Tr | Mortensen 1938 |
| *Ophiothrix spiculata* | 110 | 0.697 | P | O | Te | Rumrill & Pearse 1985, this study |
| *Ophiothrix suensonii* | 130 | 1.15 | P | O | Tr | Mladenov 1985 |
| *Ophiothrix synoecina*  **Euryalida** | 300 | 14.137 | Ov | YE | Tr | Schoppe & Holl 1994; Schoppe 1996 |
| **Asteronychidae** |  |  |  |  |  |  |
| *Asteronyx loveni* | 800 | 268.08 | (Ov) | BE | Tr/Te | Mortensen 1912 |
| **Euryalidae** |  |  |  |  |  |  |
| *Asteromopha rousseaui* | 630 | 130.924 | (Ov) | BE | Tr | Mortensen 1933c |
| **Gorgonocephalidae** |  |  |  |  |  |  |
| *Astrotoma agassizii* | 500 | 65.45 | Ov | BE | Po | Mortensen 1936 |
| *Astrothorax waitei* | 1000 | 523.599 | Ov | BE | Te/Tr | Fell 1952 |
| *Gorgonocephalus arcticus* | 300 | 14.137 | (L) | - | Po/Te | Hendler 1991 |
| *Gorgonocephalus captumedusae* | 200 | 4.189 | L | BE | Te | Mortensen 1924a, Fell 1966 |
| *Gorgonocephalus eucnemis* | 220 | 5.575 | L | - | Te | Patent 1970 |
| **Ophiacanthida** |  |  |  |  |  |  |
| **Clarkcomidae** |  |  |  |  |  |  |
| *Clarkcoma australis** | 96 | 0.463 | (P) | - | Te | This study |
| *Clarkcoma bollonsi** | 100 | 0.524 | (P) | - | Te | This study |
| *Clarkcoma canaliculata* | 266 | 10.079 | L | V | Te | Falkner et al. 2015; Hodin et al. 2019 |
| *Clarkcoma pulchra* | 290 | 12.77 | L | V | Te | Falkner et al. 2015 |
| **Ophiacanthidae** |  |  |  |  |  |  |
| *Ophiacantha bidentata* | 500 | 65.45 | Ov | BE | Te | Thorson 1936; Tyler 1980 |
| *Ophiacantha imago* | 400 | 33.51 | Ov | BE | Po | Mortensen 1920a |
| *Ophiomitrella clavigera* | 900 | 381.704 | Ov | BE | Te | Mortensen 1920a |
| *Ophiophthalmus normani* | 600 | 113.097 | (Ov) |  | Te | Rokop 1974 |
| *Ophiosabine anomala* | 500 | 65.45 | Ov | BE | Te | Mortensen 1920a |
| *Ophiosabine pentactis* | 750 | 220.893 | (Ov) | BE | Po | Hendler 1991 |
| *Ophiosabine vivipara* | 600 | 113.097 | Ov | BE | Po | Mortensen 1920, 1936 |
| **Ophiobyrsidae** |  |  |  |  |  |  |
| *Ophiophrixus spinosus* | 700 | 179.594 | Ov | BE | Te | Mortensen 1933b, Tyler 1980 |
| **Ophiocomidae** |  |  |  |  |  |  |
| *Breviturma brevipes* | 65 | 0.144 | P | O | Tr | O’Hara et al. 2019 |
| *Breviturma dentata* | 75 | 0.221 | P | O | Tr/Te | O’Hara et al. 2019 |
| *Breviturma doederleini** | 80 | 0.268 | (P) | - | Tr | O’Hara et al. 2019 |
| *Breviturma longispina** | 100 | 0.524 | (P) | - | Tr | O’Hara et al. 2019 |
| *Breviturma pusilla* | 63 | 0.131 | P | O | Tr | O’Hara et al. 2019 |
| *Ophiocoma aethiops* | 55 | 0.087 | P | O | Tr | O’Hara et al. 2019 |
| *Ophiocoma anaglyptica** | 97 | 0.478 | (P) | - | Tr | O’Hara et al. 2019 |
| *Ophiocoma echinata* | 81 | 0.278 | P | O | Tr | O’Hara et al. 2019 |
| *Ophiocoma erinaceus* | 91 | 0.395 | P | O | Tr | O’Hara et al. 2019 |
| *Ophiocoma schoenleinii* | 91 | 0.395 | P | O | Tr | O’Hara et al. 2019 |
| *Ophiocoma scolopendrina* | 100 | 0.18 | P | O | Tr | DeLaroisse et al. 2013; O’Hara et al. 2019 |
| *Ophiocomella alexandri* | 71 | 0.144 | P | O | Tr | Whitehill & Moran 2012 |
| *Ophiocomella ophiactoides* | 80 | 0.268 | P | O | Tr | Emson et al. 1985 |
| *Ophiocomella pumila* | 73 | 0.204 | P | O | Tr | Mladenov 1985 |
| *Ophiomastix annulosa* | 430 | 41.63 | L | V | Tr | O’Hara et al. 2019 |
| *Ophiomastix elegans* | 384 | 29.648 | L | V | Tr | Falkner et al. 2015; O’Hara et al. 2019 |
| *Ophiomastix macroplaca** | 100 | 0.524 | (P) | - | Tr | O’Hara et al. 2019 |
| *Ophiomastix mixta* | 335 | 19.685 | L | V | Tr | O’Hara et al. 2019 |
| *Ophiomastix occidentalis** | 66 | 0.151 | (P) | - | Tr | O’Hara et al. 2019 |
| *Ophiomastix pictum* | 419 | 38.516 | L | V | Tr | O’Hara et al. 2019 |
| *Ophiomastix venosa* | 550 | 87.114 | L | V | Tr | Fourgon et al 2005 |
| *Ophiomastix wendtii* | 100 | 0.524 | P | O | Tr | O’Hara et al. 2019 |
| *Ophiomastix endeani#** | > 350 | - | (L) | (V) | Te | Falkner et al. 2015; This study |
| **Ophiodermatidae** |  |  |  |  |  |  |
| *Bathypectinura heros* | 700 | 179.594 | Ov | BE | Tr/Te | Hendler 1991 |
| *Ophiarachnella gorgonia* | 330 | 18.817 | L | V | Tr | Cisternas & Byrne 2005 |
| *Ophiarachnella ramsayi* | 342 | 20.94 | L | V | Te | Falkner et al. 2015 |
| *Ophioderma appresum* | 286 | 12.378 | L | V | Tr | This study |
| *Ophioderma brevicaudum* | 273 | 11.494 | L | V | Tr | This study |
| *Ophioderma brevispinum* | 300 | 14.137 | L | V | Tr | Grave 1916 |
| *Ophioderma cinereum* | 348 | 22.449 | L | V | Tr | This study |
| *Ophioderma devaneyi* | 320 | 17.157 | (L) | - | Tr | Hendler & Miller 1984 |
| *Ophioderma longicaudum* | 200 | 4.189 | L | V | Tr | Fenaux 1969, 1972 |
| *Ophiopsammus maculata* | 625 | 113.097 | (Ov) | - | Te | Fell 1941 |
| **Ophiomyxidae** |  |  |  |  |  |  |
| *Ophiomyxa australis* | 250 | 8.181 | (L) | - | Te | Mortensen 1924a |
| *Ophiomyxa brevirima* | 600 | 113.097 | Ov | BE | Te | Mortensen 1924 |
| *Ophiomyxa flaccida* | 880 | 356.818 | Ov | BE | Tr | Hendler & Littman 1986 |
| *Ophiomyxa vivipara* | 500 | 65.45 | Ov | BE | Po | Mortensen 1920a |
| *Ophiurochaeta littoralis* | 680 | 164.636 | Ov | BE | Tr | Byrne 1991 |
| **Ophiopezidae** |  |  |  |  |  |  |
| *Ophiopeza spinosa* | 300 | 14.137 | Ov | BE/V | Tr | Byrne et al 2008 |
| **Ophiopteridae** |  |  |  |  |  |  |
| *Ophiopteris antipodum* | 100 | 0.524 | (P) | - | Te | Mortensen 1924a |
| *Ophiopteris papillosa* | 99 | 0.508 | P | O | Te | Rumrill & Pearse 1985; This study |
| **Ophiotomidae** |  |  |  |  |  |  |
| *Ophiocomina nigra* | 90 | 0.382 | P | O | Te | Narasimhamurti 1933; O’Hara et al. 2019 |
| **Ophioscolecida** |  |  |  |  |  |  |
| **Ophioscolecidae** |  |  |  |  |  |  |
| *Ophioscolex glacilis* | 510 | 69.456 | Ov | YE | Te | Thorson 1936 |
| **Ophiurida** |  |  |  |  |  |  |
| **Ophiomusaidae** |  |  |  |  |  |  |
| *Ophiomusa lymani* | 400 | 50.965 | (L) | - | Tr/Te | Tyler & Gage 1979,1980 |
| **Ophiopyrgidae** |  |  |  |  |  |  |
| *Amphiophiura rowetti* | 500 | 65.45 | Ov | BE | Po | Mortensen 1936, Hendler 1991 |
| *Ophioplinthus gelida* | 300 | 14.137 | (L) | - | Po | Mortensen 1936 |
| *'Ophiura' flexibilis* | 300 | 14.137 | (L) | - | Po | Mortensen 1936 |
| *Ophiuroglypha robusta* | 400 | 33.51 | Ov | BE | Po | Mortensen 1936 |
| *Stegophiura nodosa* | 600 | 113.097 | Ov | BE | Po | Mortensen 1920a |
| *Stegophiura vivipara* | 400 | 33.51 | Ov | BE | Te | Matsumoto 1915; Mortensen 1920a |
| **Ophiosphalmidae** |  |  |  |  |  |  |
| *Ophiosphalma spinigerum* | 350 | 22.449 | Ov | BE | Te | Hendler 1991 |
| **Ophiuridae** |  |  |  |  |  |  |
| *Ophiocten gracillis* | 110 | 0.697 | P | - | Te | Gage & Tyler 1981; Tyler & Gage 1982; Tyler et al. 1983 |
| *Ophiocten hastatum* | 140 | 1.43 | P | O | Te | Gage et al. 2004 |
| *Ophiocten sericeum* | 170 | 2.572 | P | O | Te | Thorson 1934,1936 |
| *Ophionotus victoriae* | 150 | 1.767 | P | O | Po | Mortensen 1936; Grange et al. 2004 |
| *Ophiura albida* | 120 | 0.905 | P | O | Te | Chadwick 1914, Tyler 1977 |
| *Ophiura ljungmani* | 100 | 0.697 | P | O | Te | Tyler & Gage 1979, 1980 |
| *Ophiura ophiura* | 110 | 0.697 | P | O | Te | Mortensen 1931, Thorson 1946, Tyler 1977 |
| *Ophuira sarsi* | 110 | 0.697 | P | O | Te | Takeda et al. 2004 |
| *Ophiomastus meriodionalis* | 400 | 33.51 | Ov | BE | Te | Mortensen 1936 |

**References**

Allen JD, Podolsky RD. 2007. Uncommon diversity in developmental mode and larval form in the genus *Macrophiothrix* (Echinodermata: Ophiuroidea)*.* Mar Biol 151:85-97.

Bowmer T. 1982. Reproduction in *Amphiura filiformis* (Echinodermata: Ophiuroidea): seasonality in gonad development. Mar Biol 9:281-290.

Brogger MI, Martinez MI, Zabala S, Penchaszadeh PE. 2013. Reproduction of *Ophioplocus januarii* (Echinodermata: Ophiuroidea): a continuous breeder in northern Patagonia, Argentina. Aquatic Biology. 19:275-285.

Byrne M. 1989. Ultrastructure of the ovary and oogenesis in the ovoviviparous brittlestar *Ophiolepis paucispina* (Echinodermata: Ophiuroidea). Biol Bull 176:79-95.

Byrne M. 1991. Reproduction, development and population biology of the Caribbean ophiuroid *Ophionereis olivacea* a protandrous hermaphrodite that broods its young. Mar Biol 111:387-399.

Byrne M, Cisternas P, O’Hara, TO. 2008. Brooding of pelagic-type larvae in *Ophiopeza spinosa*: reproduction and development in a tropical ophiodermatid brittlestar. Invert Biol 127:98–107.

Chadwick HC. 1914. Echinoderm larvae of Port Erin. Proc Trans Liverpool Biol Soc. 28:467-498.

Cisternas P, Byrne M. 2005. Larval development in the ophiuroid *Ophiarachnella gorgonia* (Ophiodermatidae): evidence for developmental heterochronies between species with Type I and type II development. Can. J. Zool. 83:1067-1079.

Delaroisse, J, Fourgon D, Eeckhaut I. 2013. Reproductive cycles and recruitment in *Ophiomastix venosa* and *Ophiocoma scolopendrina*, two co-existing tropical ophiuroids from the barrier reef of Toliara (Madagascar). Car Biol Mar 54:593-603.

Emlet RB. 2006. Direct development of the brittle star *Amphiodia occidentalis* (Ophiuroidea, Amphiuridae) from the northeastern Pacific Ocean. Invert Biol 125:154-171.

Emson R. H., Mladenov P. V, Wilkie IC. 1985. Patterns of reproduction in small Jamaican brittlestars: fission and brooding predominate. NOAA Symp. Ser. Undersea. Res. 33:87-100.

Falkner I, Sewell MA, Byrne M. 2015. Evolution of maternal provisioning in ophiuroid echinoderms: characterisation of egg composition in planktotrophic and lecithotrophic developers. Mar Ecol Prog Ser 525:1–13

Fell HB. 1941. The direct development of a New Zealand ophiuroid. Q. J. Microsc. Sci. 82:377-441.

Fell HB. 1952. Echinoderms from Southern New Zealand. Zool Publ.Victoria Univ.Wellington. 18:1-37.

Fell HB. 1966. The ecology of ophiuroids. In: Physiology of Echinodermata, Boolootian RA, editor, Wiley, New York, pp 129-143

Fenaux L. 1963. Note preliminaire sur le developpement larvaire de *Amphiura chiajei* (Forbes). Vie Milieu 14:91-96.

Fenaux L. 1969. Le development larvaire chez *Ophioderma longicauda* (Retzius). Cah Biol Mar 59-62.

Fenaux L. 1970. Maturation of the gonads and seasonal cycle of the planktonic larvae of hte ophiuroid *Amphiura chiajei* Forbes. Biol Bull 138:262-271.

Fenaux L. 1972. Evolution saisonniere des gonades chez l’ophiure *Ophioderma longicauda* (Retzius), Ophiuroidea. Int Revue Ges Hydrobiol 57: 257-262.

Fourgon D, Eeckhaut I, Vatilingon D, Jangoux M. 2005. Lecithotrophic development and metamorphosis in the Indo-West Pacific brittle star *Ophiomastix venosa* (Echinodermata: Ophiuroidea). Invert. Reprod. Dev. 47:155-165.

Gage JD, Anderson RM, Tyler PA, Chapman R, Dolan E. 2004. Growth, reproduction and possible recruitment variability in the abyssal brittle star *Ophiocten hastatum* (Ophiuroidea : Echinodermata) in the NE Atlantic. Deep-sea Research I. 51:849-864.

Gage JD, Tyler PA. 1981. Non-viable seasonal settlement of larvae of the upper bathyal brittle star *Ophiocten gracilis* in the Rockall Trough Abyssal. Mar Biol 4:153-161.

Grange LJ, Tyler PA, Peck LS, Cornelius N. 2004. Long-term interannual cycles of the gametogenic ecology of the Antarctic brittle star *Ophionotus victoriae*. Mar Ecol Prog Ser 278:141-155.

Grave C. 1916. *Ophiura brevispina*. II. An embryological contribution and a study of the effect of yolk substance upon development and developmental processes. J Morphol 27:413-451.

Heatwole DW, Stancyk SE. 1982. Spawning and functional morphology of the reproductive system of in the ophiuroid, *Hemipholis elongata*. In: Lawrence JM, editor. Echinoderms: Proceedings of International Conference, Tampa Bay. Rotterdam: Balkema. p. 469-474.

Hendler G. 1973. Northwest Atlantic amphiurid brittlestars, *Amphioplus abditus* (Verrill), *Amphioplus* *macilentus* (Verrill), *Amphiolpus sepultus* n. sp. (Ophiuroidea: Echinodermata): systematics, zoogreography, annual periodicities and larval adaptations. PhD. Thesis, Univ Connecticut, Storrs, Conn.

Hendler G. 1975. Adaptational significance of the patterns of ophiuroid development. Amer. Zool. 15:691-715.

Hendler G. 1977. Development of *Amphioplus abditus* (Verrill) (Echinodermata: Ophiuroidea): I. Larval biology. Biol Bull 152:51-63.

Hendler G. 1979a. Sex-reversal and viviparity on Ophiolepis kieri, n. sp., with notes on viviparous brittlestars from the Caribbean (Echinodermata: Ophiuroidea). Proc Biol Soc Wash 92:783–795.

Hendler G. 1979b. Reproductive periodicity of ophiuroids (Echinodermata: Ophiuroidea) on the Atlantic and Pacific coasts of Panama. In: Stancyk SE, editor. Reproductive ecology of marine invertebrates. Columbia (SC): University of South Carolina Press, p. 145–156.

Hendler G. 1982. An echinoderm vitellaria with a bilateral larval skeleton: evidence for the evolution of ophiuroid vitellariae from ophioplutei. Biol Bull 163:431-437.

Hendler G. 1991. Echinodermata: Ophiuroidea. Pp 356–479 in Reproduction of Marine Invertebrates, Vol VI. Echinoderms and Lophophorates, A. C. Giese, J. S. Pearse, V. B. Pearse, eds. Boxwood, Pacific Grove, CA.

Hendler G. 1995. New Species of brittle stars from the Western Atlantic, *Ophionereis vittata, Amphioplus sepultus*, *Ophiostigma siva*, the description of a neotype for *Ophiostigma isocanthum* (Say) (Echinodermata: Ophiuroidea*).* Contributions in Science, Natural History Museum of Los Angeles County. 458:1-19

Hendler G, Miller JE. 1984. *Ophioderma devaneyi* and *Ophioderma ensiferum*, new brittlestar species from the western Atlantic (Echinodermata: Ophiuroidea). Proc Biol Soc. Wash. 97:442-461.

Hendler G, Littman BS. 1986. The ploys of sex: relationships among the mode of reproduction, body size and habitats of coral-reef brittlestars. *Coral Reefs* 5:31-42.

Hendler G, Bundrick CJ. 2001. A new brooding brittle star from California (Echinodermata: Ophiuroidea: Amphiuridae). Contributions in Science. 486:1-11.

Hendler G, Tran LU. 2001. Reproductive biology of the deep-sea star Amphiura carchara (Echinodermata: Ophiuroidea). Mar Biol 138:113-123.

Hendler G, Miller JE, Pawson DL, Kier PM. 1995. Class Ophiuroidea. In: Sea Stars, Sea Urchins, and Allies, Echinoderms of Florida and the Caribbean. Washington: Smithsonian Institution. p. 89–180.

Hodin JA, Heyland A, Mercier A, Pernet B, Cohen DL, Hamel J-F, Allen JD, McAlister JS, Byrne M, Cisternas P, George SB. 2019. Chapter 6 - Culturing echinoderm larvae through metamorphosis. In: Foltz KR, Hamdoun A, editors. Methods in cell biology: vol. 150. Echinoderms, Part A. Cambridge (MA): Elsevier. p. 125–169.

Kitazawa C, Akahoshi S, Sohara S, Noh JT, Tajika A, Yamanaka A, Komatsu M. 2014. Development of the brittle star *Ophiothrix exigua* Lyman, 1874 a species that bypasses early unique and typical planktotrophic ophiopluteus stages. Zoomorphology 134: 93–105.

Komatsu M, Shosaku T. 1993. Development of the brittle star *Ophioplocus japonicus* H. L. Clark. Zool Sci 10:295-306.

MacBride EW. 1907. The development of *Ophiothrix fragilis*. Q J Microsc Sci 51:557-606.

Matsumoto H. 1915. A new classification of the Ophiuroidea: with descriptions of new genera and species. Proc Acad Nat Sci. Phila 67:43-92.

Mladenov PV. 1979. Unusual lecithotrophic development of the Caribbean brittle star *Ophiothrix oerstedi*. Mar Biol 55:55-62.

Mladenov PV. 1985. Development and metamorphosis of the brittle star *Ophiocoma pumila:* evolutionary and ecological implications. Biol Bull. 168:285-295.

Mortensen TH. 1893. Über *Ophiopus arcticus* (Ljungman), eine Ophiure mit rudimentaren Bursae. Z Wiss Zool 56:506-528.

Mortensen TH. 1912. Über *Asteronyx loveni*. M.Tr. Z Wiss Zool 101:264–289.

Mortensen TH. 1920a. On hermaphroditism in viviparous ophiuroids. Acta Zool 1920:1-18

Mortensen TH. 1920b. Notes on the development and larval forms of some Scandinavian echinoderms. Vidensk Medd Dan Naturhist Foren 71:133–160.

Mortensen TH. 1921. *Studies of the development and larval forms of echinoderms.* G.E.C. Gad. Copenhagen.

Mortensen TH. 1924a. Papers from Dr. TH. Mortensen’s Pacific expedition 1914-1916. XX. Echinoderms of New Zealand and the Auckland-Campbell islands. II. Ophiuroidea. Vidensk Medd Dan Naturhist Foren Kjobenhavn 76:91–178.

Mortensen TH. 1924b. Observations of some echinoderms from the Trondhjem Fjord. Kongel Norske Vidensk Selsk.Skr Trondhjem 1923:3-22.

Mortensen TH. 1931. Contributions to the study of the development and larval forms of echinoderms I-II. Kongel Danske Vidensk Selsk Skr Naturvidensk Math Afd 4:1–39.

Mortensen TH. 1933a. Papers from Dr. TH. Mortensen’s Pacific Expedition 1914-1916. LXIII. Biological observations on ophiuroids, with descriptions of two new genera and four new species. Vidensk Medd Dan Naturhist Foren 93:171–194.

Mortensen TH. 1933b. Ophiuroidea. Dan Ingolf-Exped 4:1–121.

Mortensen TH. 1933c. Studies of Indo-Pacific euryalids. Vidensk Medd Dan Naturhist Foren 96:1-75.

Mortensen TH. 1933d. Papers from Dr. TH. Mortensen’s Pacific Expedition 1914-1916. LXVI. The echinoderms of St. Helena. Vidensk Medd Dan Naturhist Foren 93:402–473.

Mortensen TH. 1936. Echinoidea and Ophiuroidea. Discovery Rep 12:199-348.

Mortensen TH. 1937. Contributions to the study of the development and larval forms of echinoderms III. Kongel Danske Vidensk Selsk Skr Naturvidensk Math Afd 7:1–65.

Mortensen TH. 1938. Contributions to the study of the development and larval forms of echinoderms IV. Kongel Danske Vidensk Selsk Skr Naturvidensk Math Afd 7:1–59.

Narasimhamurti N. 1933. The development of *Ophiocomina nigra*. Q J Microscr Sci 76:63–88.

O’Hara TD, Hugall AF, Cisternas PA, Boissin E, Bribiesca-Contreras G, Sellanes J, Paulay G, Byrne M. 2019. Phylogenomics, life history and morphological evolution of ophiocomid brittlestars. Mol Phyl Evol 130:67-80

Olsen H. 1942. Development of the brittle-star *Ophiopholis aculeata* (O. Fr. Muller) with a short note on the outer hyaline layer. Bergens Museums Aarbog Naturvideskabelig række. 6:1-107.

Patent DH. 1970. The early embryology of the basket star *Gorgonocephalus caryi* (Echinodermata, Ophiuroidea). Mar Biol 6:262-267.

Rokop FJ. 1974. Reproductive patterns in the deep-sea benthos. Science. 186:743-745.

Rumrill SS, Pearse JS. 1985. Contrasting reproductive periodicities among northeastern Pacific ophiuroids. In: Keegan BF, O’Connor BDS, editors. Echinodermata. Proceedings of the 5^th^ International Echinoderms Conference, Galway. Balkema, Rotterdam. p. 633–638.

Schoener A. 1972. Fecundity and possible mode of development of some deep-sea ophiuroids. Limnol Oceanogr 17:193-199.

Schoppe S, Holl A. 1994. *Ophiothrix* n species (Echinodermata: Ophiuroidea) from Columbia, a protandric hermaphrodite that broods its young. In: David B, Guille A, Féral JP, Roux M, editors. Echinoderms Through Time. Balkema, Rotterdam. P. 471-475

Schoppe S. 1996. *Ophiothrix synoecina* new species (Echinodermata: Ophiotrichidae) from the Caribbean coast of Columbia. Bull Mar Sci 58:429-437.

Selvakumaraswamy P, Byrne M. 2000. Reproduction, spawning, development of 5 ophiuroids from Australia and New Zealand. Invert Biol 119:394-402.

Selvakumaraswamy P, Byrne M. 2004. Metamorphosis and developmental evolution in *Ophionereis* (Echinodermata: Ophiuroidea). Mar Biol 145:87-99.

Selvakumaraswamy P, Byrne M. 2006. Evolution of larval form in ophiuroids, insights from the metamorphic phenotype of *Ophiothrix* (Echinodermata: Ophiuroidea). Evol Dev 8:183–190.

Smith JE. 1940. The reproductive system and associated organs of the brittle-star *Ophiothrix fragilis*. Quart J Microsc Sci 82: 267-309.

Stancyk, S. E. 1970. Studies on the biology and ecology of ophiuroids at Cedar key, Florida. M.Sc. Thesis, Univ. Florida, Gainesville, Florida.

Stancyk, S. E. 1973. Development of *Ophiolepis elegans* (Echinodermata: Ophiuroidea) and its implications in the estuarine environment. Mar Biol 21:7-12.

Sweet, H. C., M. C. Doolin., C. N. Yanowiak., A. D. Coots., A. W. Freyn., J. M Armstrong., B. J. Spiecker. 2019. Abbreviated development of the brooding brittle star *Ophioplocus esmarki*. Biol Bull 236:75-87.

Takeda M, Kogure Y, Yoshizawa H, Komatsu M. 2004. Development of the deep-sea ophiuroid, *Ophiura sarsi* Lutken 1855. Zool Sci 21:1270.

Thorson G. 1934. On the reproduction and larval stages of the brittle-stars *Ophiocsten sericeum* (Forbes) and *Ophiura robusta* Ayres in East Greenland. Medd Groenl 100:1-20.

Thorson G. 1936. The larval development, growth and metabolism of Arctic marine bottom invertebrates- compared with those of other seas. Medd Groenl 100:1-155.

Thorson G. 1946. Reproduction and larval development of Danish marine bottom invertebrates, with special reference to the planktonic larvae in the Sound (Øresund). Medd Komm Dan Fisk-og Havunders. Ser Plankton. 4:1-523.

Tominaga, H., Nakamura, S., Komatsu, M. 2004. Reproduction and development of the conspicuously dimorphic brittle star *Ophiodaphne formata*. Biol Bull 206:25-34.

Tyler PA. 1977. Seasonal variation and ecology of gametogenesis in the genus *Ophiura* (Ophiuroidea: Echinodermata) from the Bristol Channel. J Exp Mar Biol Ecol 30: 185-197

Tyler PA. 1980. Deep-sea ophiuroids. Oceanogr Mar Biol Ann Rev 18:125-153.

Tyler PA, Gage JD. 1979. Reproductive ecology of deep sea ophiuroids from the Rockall Trough. In: Naylor E, Hartnoll RG, editors. Cyclic phenomena in marine plants and animals. Oxford: Pergamon Press. p. 215–222.

Tyler PA, Gage JD. 1980. Reproductive patterns in deep sea ophiuroids from the North-East Atlantic. In: Jangoux M, editor. Echinoderms: present and past. Rotterdam: Balkema. p. 417–421.

Tyler PA, Gage JD. 1982. *Ophiopluteus ramosus*, the larval form of *Ophiocten gracilis* (Echinodermata: Ophiuroidea). J Mar Biol Assoc UK 62:485–486.

Tyler PA, Gage JD, Pain SL. 1983. Reproductive variability in deep-sea echinoderms and molluscs from the Rockall Trough. Oceanol Acta Spec Issue 1983:191–195.

Whitehill E. A. G., A. L. Moran. 2012. Comparative larval energetics of an ophiuroid and an echinoid echinoderm. Invert Biol 31:345–354.

Yamashita M. 1985. Embryonic development of the brittle-star *Amphipholis kochii* in laboratory culture. Biol Bull 169: 131–142.
