# Supplementary material for: Evolution of Maternal Provisioning and Development in the Ophiuroidea: Egg Size, Larval Form, and Parental Care
Source: Integr Comp Biol. 2024 May 23;64(6):1536–55. doi: 10.1093/icb/icae048 (PMC11659680; doi:10.1093/icb/icae048)
Supplement: icae048_Supplemental_Files — Figure S1. The distributions of egg volume and developmental mode in ophiuroids with egg sizes of the facultative planktotrophy included (see legend Fig. 4). Egg size in the two species with facultative planktotrophy, Amphiodia sp. (opaque) and Macrophiothrix rabdota (see Allen and Podolsky 2007; Nakata and Emlet 2023) have intermediate positions in the overall egg size distribution. [file icae048_supplemental_files.zip › icb-2024-0007-File009.docx]

**Table S2.** Examples of viviparous (matrotrophic) ophiuroids where extreme disparity between egg and offspring size indicate extensive extraembryonic nutrition supporting development. Species with this mode of provisioning were not included in the egg size frequency analysis. See Hendler (1991) for a full list and review. Also included are the two species with facultative feeding larvae. NA, not applicable.

| Species | Offspring nutrition | Egg diameter µm | Egg volume (nl) | Maximum  juvenile size, Disc diameter µm | Reference |
| --- | --- | --- | --- | --- | --- |
| *Amphipholis squamata* | Matrotrophy | 100-120 | 0.524-0.905 | 1200 µm | Byrne 1991; Hendler 1991; Walker and Lesser 1989 |
| *Ophioderma wahlbergii* | Matrotrophy | 250 | 8.181 | 9330 | Landschoff and Griffiths 2015 |
| *Ophionotus hexactis* | Matrotrophyy | 200 | 4.189 | 8300 | Turner and Dearborn 1979; Hendler 1991 |
| *Amphiodia* species opaque | Facultative feeding larvae | 140 | 1.44 | NA | Nakata and Emlet 2023 |
| *Macrophothrix rhabdota* | Facultative feeding larvae | 230 | 6.37 | NA | Allen and Podolsky 2007 |

References

Allen JD, Podolsky RD. 2007. Uncommon diversity in developmental mode and larval form in the genus *Macrophiothrix* (Echinodermata: Ophiuroidea)*.* Mar Biol 151:85-97.

Byrne M. 1991. Reproduction, development and population biology of the Caribbean ophiuroid *Ophionereis olivacea* a protandrous hermaphrodite that broods its young. Mar Biol 111:387-399.

Hendler G. 1991. Echinodermata: Ophiuroidea. In: Giese AC, Pearse JS, Pearse VB, editors. Reproduction of Marine Invertebrates, Vol VI. Echinoderms and Lophophorates. Pacific Grove, California: Boxwood. p. 356–479

Landschoff JL, Griffiths CL. 2015 Brooding behavior in the shallow-water brittle star *Ophioderma wahlbergii.* Inv Biol 134:168-179.

Nakata NN, Emlet RB. 2023. Having cake and eating too: the benefits of an intermediate larval form in a brittle star *Amphiodia* sp. opaque (Ophiuroidea). Ecol Evol 13:e10298.

Turner RL, Dearborn JH. 1979. Organic and inorganic composition of post-metamorphic growth stages of *Ophionotus hexactis* (EA Smith) (Echinodermata: ophiuroidea) during intraovarian incubation. J Exp Mar Biol Ecol. 36:41–51

Walker CW, Lesser MP. 1989. Nutrition and development of brooded embryos in the brittlestar *Amphipholis squamata*: do endosymbiotic bacteria play a role? Mar Biol 103:519-530.

.
