# Supplementary material for: Evolution of Maternal Provisioning and Development in the Ophiuroidea: Egg Size, Larval Form, and Parental Care
Source: Integr Comp Biol. 2024 May 23;64(6):1536–55. doi: 10.1093/icb/icae048 (PMC11659680; doi:10.1093/icb/icae048)
Supplement: icae048_Supplemental_Files — Figure S1. The distributions of egg volume and developmental mode in ophiuroids with egg sizes of the facultative planktotrophy included (see legend Fig. 4). Egg size in the two species with facultative planktotrophy, Amphiodia sp. (opaque) and Macrophiothrix rabdota (see Allen and Podolsky 2007; Nakata and Emlet 2023) have intermediate positions in the overall egg size distribution. [file icae048_supplemental_files.zip › icb-2024-0007-File010.pdf]

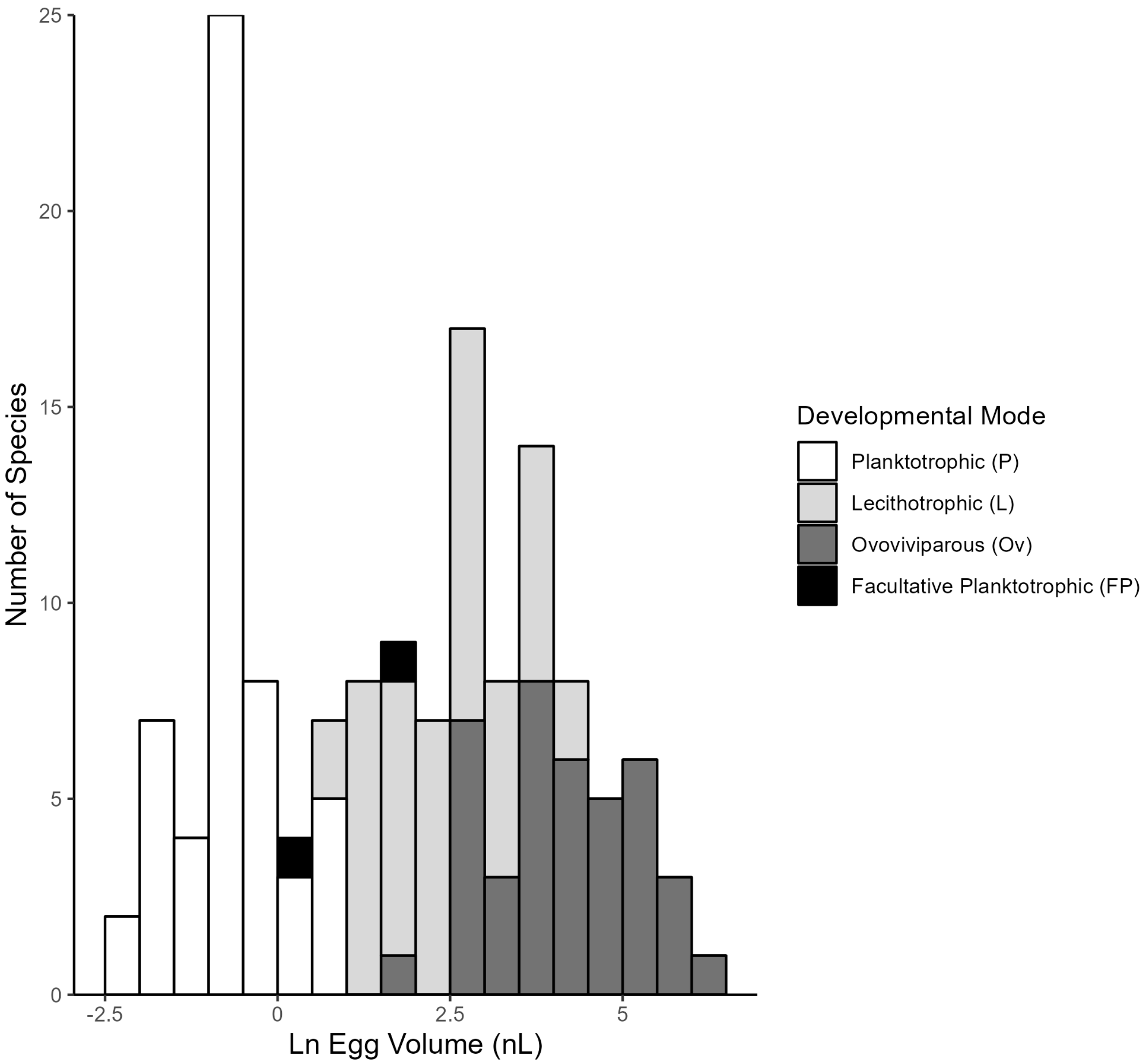

Figure S1. The distributions of egg volume and developmental mode in ophiuroids with egg sizes of the facultative planktotrophy included (see legend Fig. 4). Egg size in the two species with facultative planktotrophy, *Amphiodia* sp. (opaque) and *Macrophiothrix rabdota* (see Allen and Podolsky 2007; Nakata and Emlet 2023) have intermediate positions in the overall egg size distribution.
